# A phenotype centric benchmark of variant prioritisation tools

**DOI:** 10.1038/s41525-018-0044-9

**Published:** 2018-02-05

**Authors:** Denise Anderson, Timo Lassmann

**Affiliations:** Telethon Kids Institute, The University of Western Australia, Subiaco, WA 6008 Australia

**Keywords:** Disease genetics, Genetic markers, Medical genomics, Data mining, Genome informatics

## Abstract

Next generation sequencing is a standard tool used in clinical diagnostics. In Mendelian diseases the challenge is to discover the single etiological variant among thousands of benign or functionally unrelated variants. After calling variants from aligned sequencing reads, variant prioritisation tools are used to examine the conservation or potential functional consequences of variants. We hypothesised that the performance of variant prioritisation tools may vary by disease phenotype. To test this we created benchmark data sets for variants associated with different disease phenotypes. We found that performance of 24 tested tools is highly variable and differs by disease phenotype. The task of identifying a causative variant amongst a large number of benign variants is challenging for all tools, highlighting the need for further development in the field. Based on our observations, we recommend use of five top performers found in this study (FATHMM, M-CAP, MetaLR, MetaSVM and VEST3). In addition we provide tables indicating which analytical approach works best in which disease context. Variant prioritisation tools are best suited to investigate variants associated with well-studied genetic diseases, as these variants are more readily available during algorithm development than variants associated with rare diseases. We anticipate that further development into disease focussed tools will lead to significant improvements.

## Genomic analysis: Tools for prioritizing gene variants impacted by disease context

The performance of software tools used to distinguish disease-causing genetic variants depends on the type of disease under investigation. Denise Anderson and Timo Lassmann from the Telethon Kids Institute in Subiaco, Australia, compared 24 software tools commonly used to narrow down DNA variants found in next-generation sequencing data to those likely to cause a particular disease. By looking at more than 4000 disease phenotypes, the researchers found that the different prioritisation tools, owing to their different methodologies and algorithms, varied in their ability to discriminate between pathogenic and benign gene variants. The top-performing tools all used machine-learning techniques and worked best in cases of well-studied genetic diseases. The findings highlight the need for additional disease-focused tool development, and offer a resource to help researchers decide which approach to use in different disease contexts.

## Introduction

Dramatic progress in next-generation sequencing technologies has led to whole-genome sequencing (WGS) and whole-exome sequencing (WES) becoming valuable tools when attempting to diagnose patients with genetic diseases.^1^ Despite this progress, many patients remain undiagnosed even after sequencing efforts.^2^ WGS has many advantages over WES including coverage of non-coding regions, better coverage of exonic regions and the ability to detect copy number variation, all of which lead to better diagnostic yield.^3–6^ Despite this, WES is more widely used than WGS, due to WGS being cost prohibitive for some applications, however a shift in this balance is expected as the cost of WGS continues to fall.^7^ WES typically discovers between 60,000 and 100,000 variants per individual.^8^ The vast majority of those are benign or unrelated to the observed disease phenotype of the patient. Discovering causative variants from this large background is extremely challenging, exacerbated by the presence of around 100 loss of function variants unrelated to the presented disease phenotype.^9^ Furthermore, variant detection pipelines require expert calibration to ensure optimal results for each sequencing platform.^10^ Variant prioritisation tools assist in the discovery of putative causal variants for follow-up. There are many such tools available, making it difficult for the end user to select the most appropriate tool for their particular study. Previous work demonstrated that the performance of these tools varies widely.^11–17^

Broadly speaking, variant prioritisation tools can be classified into four categories: (1) tools exploiting the fact that evolutionarily conserved regions are likely to be functional and (2) tools that predict the effect of variants on protein sequence and structure and (3) machine learning classifiers of variant pathogenicity that incorporate conservation scores, protein functional prediction scores and other functional genomic data as predictor variables and (4) ensemble methods that are similar to machine learning classifiers but additionally include functional predictions from a number of variant prioritisation tools.

We hypothesise that the performance of tools varies by disease phenotype. To test this hypothesis we created an automatic pipeline to generate disease stratified benchmark data sets for variant prioritisation tools. In a three step process, we used (a) the Human Phenotype Ontology (HPO) resource to obtain terms for human phenotypic abnormalities associated with disease,^18^ (b) linked these to the associated genes using the Phenolyzer tool^19^ and finally retrieved all known pathogenic variants in these genes from ClinVar.^20^ In total we tested 24 variant prioritisation tools across 4026 disease phenotypes.

## Results

### Categorisation of variant prioritisation tools

The latest publication of dbNSFP^21^ categorises variant prioritisation tools as conservation scores, functional prediction scores, general prediction scores and ensemble scores. We used six conservation scores (GERP++, phastCons100way-vertebrate, phastCons20way-mammalian, phyloP100way-vertebrate, phyloP20way-mammalian and SiPhy), nine functional prediction scores (FATHMM, LRT, MutationAssessor, MutationTaster, PolyPhen2-HDIV, PolyPhen2-HVAR, PROVEAN, SIFT and VEST3), six general prediction scores (CADD, DANN, Eigen-PC, fathmm-MKL, fitCons-i6 and GenoCanyon) and three ensemble scores (M-CAP, MetaLR and MetaSVM).

### Distribution of genes and pathogenic variants in disease stratified gene panels

We analysed 11,722 HPO ‘Phenotypic abnormality’ terms and found that 6627 of these had at least one gene returned by Phenolyzer. We refer to these gene lists as gene panels. Many of the HPO terms had no gene panels because lower levels of the ontology are very specific in regards to the ‘Phenotypic abnormality’. For example, HP:3000079 is the term for ‘Abnormality of mandibular symphysis’ and this term had no genes returned by Phenolyzer but the ancestor of HP:3000079 (HP:0000924 ‘Abnormality of the skeletal system’) had 2743 genes returned by Phenolyzer. Table 1 shows the distribution of the number of genes returned by Phenolyzer for the HPO terms, based on the six gene panel types outlined in Methods (Performance evaluation). The distribution of the number of genes per HPO term shifts as expected, toward each term being associated with fewer genes as the confidence threshold (stringency) is increased. When using the expanded gene panels with no score threshold, the number of genes returned for each HPO term was very high, with 5727 terms associated with more than 1000 genes. An example of this can be seen when querying the Phenolyzer web server with ‘autism’, where the gene panel contains 474 genes versus the expanded gene panel with 18,249 genes.

**Table 1:** Distribution of the number of genes returned by Phenolyzer

Phenolyzer gene list type	1–10	11–50	51–250	251–500	501–1000	>1000
Gene panels threshold = 0	3637	1450	1041	259	152	88
Gene panels threshold = 0.25	4807	1551	268	1	0	0
Gene panels threshold = 0.5	6108	515	4	0	0	0
Extended gene panels threshold = 0	214	63	204	182	237	5727
Extended gene panels threshold = 0.25	4696	1344	388	73	68	58
Extended gene panels threshold = 0.5	5874	683	70	0	0	0

There were 6113 unique gene symbols across all the gene panels returned by Phenolyzer, and 24,632 unique gene symbols (82% of all protein-coding genes) across all the expanded gene panels. These were used to query dbNSFP by HGNC symbol for annotated variants within these genes. dbNSFP did not contain variant annotation for 970 of the 6113 genes (16%) from the gene panels, nor 7281 of the 24,632 genes (30%) from the expanded gene panels. Using Entrez Gene annotation (downloaded from ftp://ftp.ncbi.nih.gov/gene/DATA/GENE_INFO/Mammalia/Homo_sapiens.gene_info.gz on 16 August, 2016) we found that the 970 genes without dbNSFP annotation were primarily probable genes of unknown type (62.5%), pseudogenes (18.2%) and non-coding RNA (10.3%), and the 7281 genes were primarily non-coding RNA (53.5%), pseudogenes (26.1%) and probable genes of unknown type (9.7%). In summary, we were able to retrieve dbNSFP variant annotation for 5143 genes across the gene panels and 17,351 genes across the expanded gene panels.

Next we filtered the dbNSFP variant annotation to retain ClinVar pathogenic variants. Of the 5143 annotated genes for the gene panels, only 2438 genes contained previously described pathogenic variants (*n* = 22,941) and of the 17,351 annotated genes for the expanded gene panels, only 2930 genes contained pathogenic variants (*n* = 24,792). When restricting this to a complete case analysis (i.e., where all tools are required to have a score for each variant) the number of pathogenic variants reduces to 11,284 for the gene panels and 12,311 for the expanded gene panels (Table 2). As expected, the number of pathogenic variants per HPO term decreases as the confidence threshold is increased. The expanded gene panel with no score threshold shows the same outlying distribution seen in Table 1, due to the high number of genes returned by Phenolyzer for each HPO term. Hence, to use results from the expanded gene panel would involve choosing a score threshold to increase the stringency of genes associated with HPO terms, and for our purposes it would be difficult to choose a single score threshold to apply to all terms. The same reasoning applies to the gene panels, where we have chosen to use results with no score threshold given the difficulty in choosing a score threshold to use across all HPO terms.

**Table 2:** Distribution of the number of ClinVar pathogenic variants returned by dbNSFP for the 11,722 HPO Phenotypic abnormality terms

Phenolyzer gene list type	0	1–10	11–50	51–250	251–500	501–1000	>1000
Gene panels threshold = 0	5657	1219	1611	1680	617	445	493
Gene panels threshold = 0.25	5848	1556	1974	1746	413	148	37
Gene panels threshold = 0.5	6015	1957	2305	1354	85	6	0
Extended gene panels threshold = 0	5217	100	105	101	107	194	5898
Extended gene panels threshold = 0.25	5838	1536	1913	1637	385	189	224
Extended gene panels threshold = 0.5	6006	1898	2227	1370	156	62	3

Given that we aimed to assess performance using both the area under the receiver operating characteristic curve (auROC) and the area under the precision-recall curve (auPRC), we further filtered the HPO terms to ensure each variant prioritisation tool had scores for at least 25 ClinVar pathogenic variants. This number of variants results in an acceptable 95% confidence interval width at an auROC of 0.7 (95% CI: 0.58–0.82). Filtering reduced the number of HPO terms we investigated from 6065 to 4026 for the complete case analysis, and from 6421 to 4108 for the analysis using all pathogenic variants. In summary, we chose to use Phenolyzer gene panels with no score threshold when assigning disease genes to each HPO term. Further to this, we required each HPO term to have variant prioritisation tool scores for at least 25 ClinVar pathogenic variants.

### Missing data across variant prioritisation tools

Variant prioritisation tools do not always provide scores for every variant contained in dbNSFP. To assess missing data we used the HPO terms filtered to have at least 25 ClinVar pathogenic variants (*n* = 4108). For each tool, the proportion of pathogenic variants with missing scores across these HPO terms differs (Supplementary Figure 1). Many of the tools have complete variant scores across most HPO terms but a number of tools (FATHMM, LRT, M-CAP, MetaLR, MetaSVM, MutationAssessor, PolyPhen2-HDIV, PolyPhen2-HVAR, PROVEAN, SIFT and VEST3) have missing scores for a significant proportion of the variants (>20%) for hundreds of the terms. We also found that M-CAP had a much higher percentage of missing data across the benign variants (38%) when compared to all other variant prioritisation tools where the percentage of missing data ranged between 0 and 11%. These results show that tools do show large differences in the amount of missing scores across HPO terms. Subsequent main results are based on the complete case analysis so that assessment of tool performance is unaffected by missing data. We used Variant Effect Predictor (Ensembl release 90—August 2017)^22^ to annotate all pathogenic variants included in the complete case analysis (*n* = 11,284) and found that almost all were classified as missense variants (94.4%). The remaining variant classifications were splice region (3.8%), stop gain (2.9%), synonymous (1.6%), stop lost (0.9%), NMD transcript (0.2%) and stop retained (0.1%) [Note: a single variant can receive more than one classification, therefore percentages will not sum to 100%].

### Characteristics of Phenolyzer genes

We investigated the dbNSFP gene annotations describing the characteristics of gene panels returned by Phenolyzer. The first measure we used was the predicted probability of gene haploinsufficiency,^23^ where the higher the predicted probability of haploinsufficiency, the less likely the gene will be functional with only one working copy. Hence, dominant genetic disorders tend to be associated with haploinsufficiency. Supplementary Figure 2 shows the distribution of this measure for all genes (*n* = 17,082) and for the Phenolyzer genes (*n* = 4679). Phenolyzer genes do show a shift toward higher probabilities of haploinsufficiency when compared to the distribution of probabilities for all genes, reflecting the enrichment of dominant genetic disorders amongst the HPO terms. The second measure we used was the predicted probability of recessive disease causation^9^ and Supplementary Figure 3 shows the distribution for all genes (*n* = 14,142) and for the Phenolyzer genes (*n* = 4338). A shift toward higher probabilities of recessive disease causation is observed for the Phenolyzer genes due to enrichment of recessive genetic disorders across the HPO terms. The third measure we used was residual variation intolerance scores (RVIS),^24^ where higher scores indicate greater tolerance of the gene to mutational burden. Supplementary Figure 4 shows the distribution of RVIS percentile ranks for all genes (*n* = 16,956) and for the Phenolyzer genes (*n* = 4774). Phenolyzer genes show a shift toward being less tolerant to mutational burden. This is due to the enrichment of genes for Mendelian diseases and genes for disease types that are intolerant to mutational burden. The fourth measure we used was LoFtool gene intolerance scores,^25^ where lower scores indicate greater gene intolerance to functional change. Supplementary Figure 5 shows the distribution of these scores for all genes (*n* = 14,515) and for Phenolyzer genes (*n* = 4285). Phenolyzer genes show a shift toward being more intolerant to functional change due to the enrichment of genes associated with disease. Hence, we find that the genes returned by Phenolyzer are enriched in genes responsible for Mendelian diseases, including dominant and recessive disorders. This is due to Phenolyzer‘s use of Mendelian disease databases as the main source of gene–disease associations.

We also assessed similarity of gene panels across the HPO terms. Supplementary Figure 6 shows a heatmap of the Jacard index for all pairs of gene panels across the HPO terms used in the complete cases analysis (*n* = 4026). Overall similarity is low (Jaccard index <0.2; >98% of all pairwise comparisons) with very few comparisons showing moderate (Jaccard index 0.4–0.6; <1% of all pairwise comparisons) or strong similarity (Jaccard index >0.8; <1% of all pairwise comparisons).

### Performance of variant prioritisation tools

**Fig. 1:**
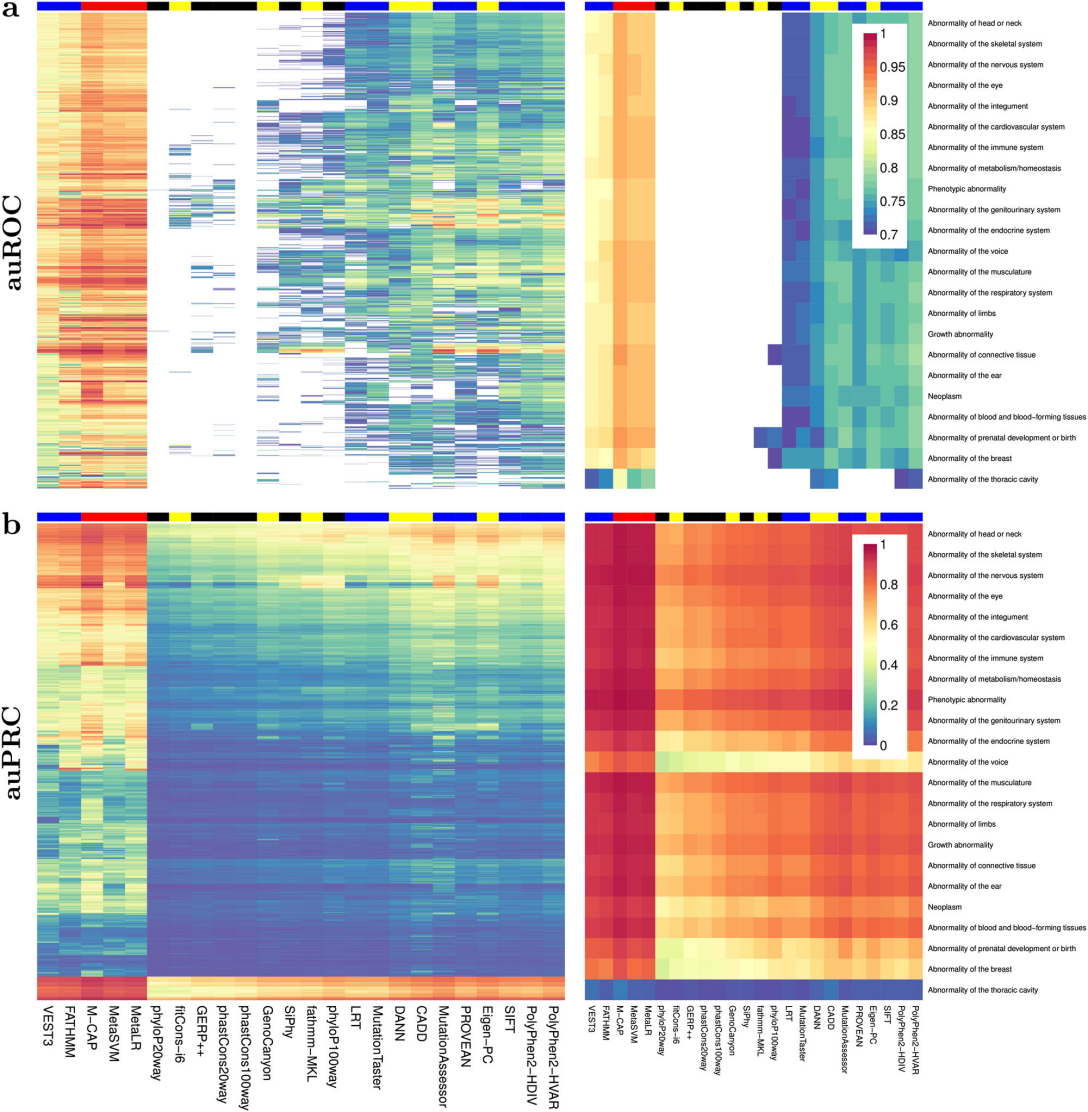
Heatmaps showing auROC (**a**) and auPRC (**b**) values for the 4026 HPO ‘Phenotypic abnormality’ terms when using Phenolyzer gene panels with no score threshold. Right-hand plots show the top level ontology (HP:0000118 ‘Phenotypic abnormality’) and broad child terms of ‘Phenotypic abnormality’. Left-hand plots show the remaining HPO terms not plotted in the right-hand plots. Colour coding of columns represents the score type for each variant prioritisation tool where black = conservation scores, red = ensemble scores, blue = functional prediction scores and yellow=general prediction scores. The heatmap colour scale of the auROC (**a**) values has been adjusted to highlight moderate to strong performance by only colour coding auROC values greater than or equal to 0.7

The overall performance of tools varies across HPO terms (Fig. 1 and Supplementary Figures 7–11). For both the auROC and the auPRC, the top performing cluster of tools includes the three ensemble scores (M-CAP, MetaLR and MetaSVM) and two functional prediction scores (FATHMM and VEST3). These five tools have high auROC values across most of the HPO terms but for the auPRC performance ranges from poor to strong. The six conservation scores (GERP++, phastCons100way-vertebrate, phastCons20way-mammalian, phyloP100way-vertebrate, phyloP20way-mammalian and SiPhy) are in the lowest performing clusters of tools for both the auROC and the auPRC.

Heatmaps of performance when analysing all variants show that the aforementioned tools remain top performers, but two general prediction scores (CADD and Eigen-PC) are also amongst the top performing cluster of tools for the auPRC (Supplementary Figures 12–17). The conservation scores remain amongst the lowest performers, but phastCons100way-vertebrate, phyloP100way-vertebrate and SiPhy show stronger performance than GERP++, phastCons20way-mammalian and phyloP20way-mammalian for the auROC.

### Performance of variant prioritisation tools by number of pathogenic variants

Next, we investigated whether performance depends on the number of available pathogenic variants. For all tools it is clear that the auROC is variable when the number of pathogenic variants is low (Supplementary Figure 18). The auROC stabilises to a constant value as the number of pathogenic variants increases. Strong performing tools based on the auROC versus number of pathogenic variants are FATHMM, M-CAP, MetaLR, MetaSVM and VEST3 where the bulk of auROC values are above 0.8.

The same trend can be observed for the auPRC (Supplementary Figure 19). However, for most tools strong performance (auPRC >0.8) is only achieved when there are many thousands of pathogenic variants. The strongest performing tools based on the auPRC versus number of pathogenic variants are FATHMM, M-CAP and MetaLR, where there is more of a shift in the distribution toward the top left of the plots when compared to other tools (indicating better overall performance across varying numbers of pathogenic variants).

### Performance of top variant prioritisation tools across specific HPO phenotypic abnormalities

Here we examined performance of the top performing tools in different disease contexts. We focussed on the auPRC given that this measure is more sensitive to the number of false positives (FPs) and therefore more relevant to the clinical setting. We considered six top level HPO terms and their descendant terms. The six top level terms are Abnormality of metabolism/homeostasis (HP:0001939), Abnormality of the cardiovascular system (HP:0001626), Abnormality of the immune system (HP:0002715), Abnormality of the musculature (HP:0003011), Abnormality of the nervous system (HP:0000707) and Abnormality of the respiratory system (HP:0002086).

**Fig. 2:**
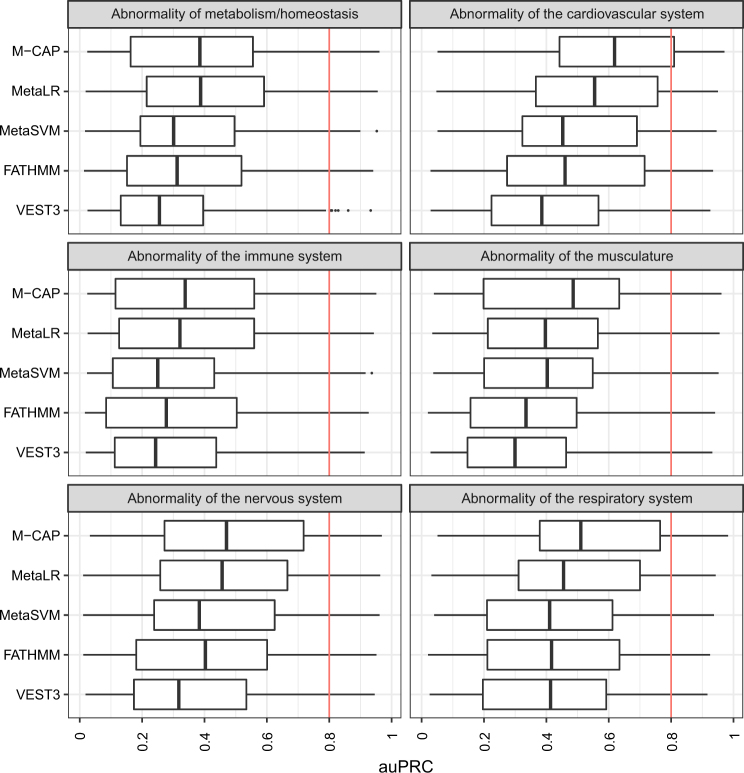
Boxplots showing the auPRC values across the top performing variant prioritisation tools for selected HPO ‘phenotypic abnormality’ terms. The vertical red line indicates a strong performance value of 0.8

Despite being the top performers, all five tools showed weak to moderate performance (0.2 < auPRC < 0.6) for most descendant terms of the six top level HPO terms (Fig. 2). M-CAP shows the strongest performance across all top level HPO terms and descendants, except ‘Abnormality of metabolism/homeostasis’ where MetaLR is the best performer. For the five HPO terms and descendants where M-CAP is the top performer, MetaLR mirrors the performance of M-CAP albeit being slightly less accurate. For all tools, best performance is seen for ‘Abnormality of the cardiovascular system’ as evidenced by the shift towards higher auPRC values when compared to the other HPO terms. Worst performance is seen for ‘Abnormality of the immune system’ where there is a shift towards lower auPRC values when compared to the other HPO terms.

For the auROC most top tools perform strongly (auROC >0.8) for most descendant terms of the six top level HPO terms, though VEST3 shows weaker performance than the other tools (Supplementary Figure 20). ‘Abnormality of the immune system’ shows the widest interquartile range across tools when compared to the other HPO terms.

Strikingly, we discovered that the performance of tools depends on the disease phenotype, even when the broadest terms are used. This suggests that depending on observed disease phenotype, different tools should be used to discover causative variants. To further explore this, we investigated HPO terms where the top performing tools show discrepancies in performance.

### Discrepancies in performance across top variant prioritisation tools

**Fig. 3:**
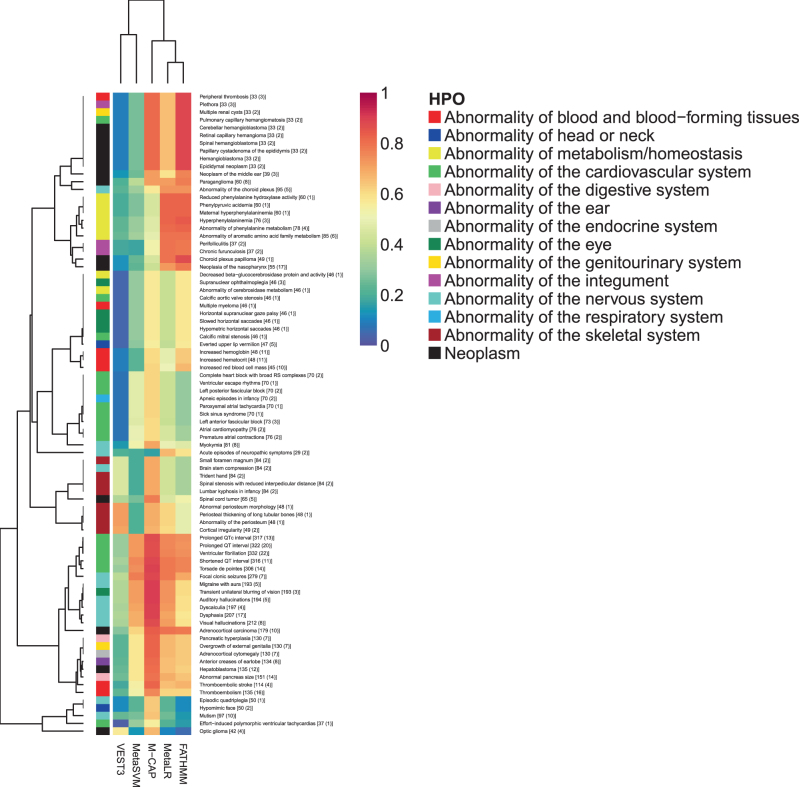
Heatmap showing auPRC for HPO ‘Phenotypic abnormality’ terms where top performing variant prioritisation tools differ by greater than 0.5. Colour coding of rows is by the parent HPO term. Row annotation includes term and [Number of ClinVar pathogenic variants (number of genes returned by Phenolyzer)]

To explore the performance differences amongst the five best tools, we plotted the 83 HPO terms where the range in auPRC values across the top tools is greater than 0.5 (Fig. 3). The terms are grouped under their parent term, and it can be seen that in most cases FATHMM, M-CAP and MetaLR show superior performance to MetaSVM and VEST3 for ‘Neoplasm’ and ‘Abnormality of metabolism/homeostasis’ terms. For ‘Abnormality of the cardiovascular system’ terms, VEST3 shows poor to weak performance whereas the other four tools show moderate to strong performance. Similarly for ‘Abnormality of the skeletal system’ terms, MetaSVMs weak performance contrasts the moderate to strong performance of the other four tools. There is variable performance across the five tools when considering ‘Abnormality of the nervous system’ terms, with M-CAP being the strongest performer for most terms, but also being one of the weakest performers for one term.

For the auROC, top performing tools do not generally show large discrepancies across the HPO terms. There are 23 terms where the range in auROC values across the top tools is greater than 0.2 (Supplementary Figure 21). Most of these terms are due to FATHMM, M-CAP, MetaLR and MetaSVM showing stronger performance than VEST3 in identifying a small number of pathogenic variants from a small number of genes. This is likely due to the FATHMM algorithm where the weighting scheme leads to ‘type 2 circularity’. This occurs when a variant is more likely to be predicted as pathogenic if other variants in the same protein are also predicted to be pathogenic^14^ (M-CAP, MetaLR and MetaSVM use FATHMM scores in their algorithms).

Top performing tools show discrepancies in the auPRC, and the pattern of discrepancies differs by HPO broad phenotype terms. We found that discrepancies in the auROC is primarily due to the FATHMM weighting scheme. Expanded results for discrepancies in performance can be found in Supplementary Tables 1 and 2. Supplementary Table 1 lists the 549 HPO terms where the range in auPRC values is greater than 0.3 and Supplementary Table 2 lists the 191 terms where the range in auROC values is greater than 0.15.

### HPO phenotypic abnormality terms where all top performing variant prioritisation tools perform strongly or poorly

In addition to the tables provided for discrepancies in performance across the top variant prioritisation tools, we also provide tables where all top tools perform strongly or poorly. These tables identify HPO terms where top tools can be used interchangeably. Supplementary Table 3 lists the 389 HPO terms where all top tools have strong auROC values (>0.9). Supplementary Table 4 lists the 515 HPO terms where all top tools have moderate to strong auPRC values (>0.7) and this tends to occur for terms which are associated with thousands of pathogenic variants (Supplementary Figure 19). There are no HPO terms where all top tools perform poorly for the auROC (<0.5). Supplementary Table 5 lists the 387 HPO terms where all top tools perform poorly for the auPRC (<0.2) and this is primarily for terms with a small number of pathogenic variants (<200).

### Recommended use of the performance results for the top variant prioritisation tools

We produced easily searchable and filterable HTML tables of the performance results for the top five tools (Supplementary Files 1 and 2). In practice, we would recommend firstly querying the tables for a HPO term of interest and considering whether any of the top tools perform adequately. Good performance would require an auROC of at least 0.8, while the auPRC should be greater than the ratio of pathogenic to benign variants. If performance is found to be adequate then the best performing tool of the five should be used for variant annotation. For users who would prefer to implement a consensus strategy for variant annotation, we would suggest using the top two or three performing tools of the five.

### Discussion

We found that performance of variant prioritisation tools does differ by disease phenotype. An example of this is seen in Fig. 2 where tools perform better for HPO terms associated with ‘Abnormality of the cardiovascular system’ versus ‘Abnormality of the immune system’. Differences are due to the number of pathogenic variants associated with each HPO term, as performance is dependent on the ratio of pathogenic to benign variants (Supplementary Figures 18 and 19). Current state of knowledge regarding genetic causes of particular disease phenotypes also contributes to differences in performance. Diseases where causal gene variants are well characterised will be overrepresented in the set of pathogenic variants used for tool training in comparison to less understood diseases. Hence variant prioritisation using prediction tools alone is best suited to well-studied genetic diseases involving a large number of causal variants. Other scenarios will require supplementation of tool scores with clinical knowledge, additional data and filtering strategies to better prioritise variants.

Variant prioritisation tools vary in their ability to discriminate between pathogenic and benign variants. This is primarily due to differing methodologies used by the tools to score variant pathogenicity. We find that the best performing tools (FATHMM, M-CAP, MetaLR, MetaSVM and VEST3) employ machine learning techniques and have markedly superior performance when compared to conservation-based scores (Fig. 1). Conservation scores consider a single factor contributing to the potential for variant pathogenicity (i.e., conservation of the genomic region), whereas machine learning algorithms incorporate a greater range of predictor variables. This additional information adds to the sensitivity of the classifier because region conservation alone does not fully explain variant pathogenicity. It is important to note that tools assessed in this study may have been trained on the pathogenic variants used in our analyses. This will result in optimistic auROC and auPRC values. We made the pragmatic decision to include all variants regardless of whether they may have been used for training. This is warranted given that our aim is to assess performance of tools ‘out of the box’ across phenotypes so we can provide advice to the end user. All tools show poor performance in identifying a small number of pathogenic variants from a large number of benign variants (Supplementary Figure 19), highlighting the need for further development in the field. Advances will occur through dynamic incorporation of increasing amounts of publicly available data and by building classifiers that are disease specific.^26^

It is important to be aware that some variant prioritisation tools will not have scored all variants of interest. This is due to some tools focussing on well characterised transcripts, rather than attempting to score all possible non-synonymous single-nucleotide variants (nsSNVs) in the genome.^12^ We conducted a complete case analysis so that comparisons between tools were unaffected by missing data. However, when attempting to prioritise variants, more complete data is obviously preferred. For some HPO ‘Phenotypic abnormality’ terms, the amount of missing data is quite high (> 20%) for some tools, including our top performers (Supplementary Figure 1), and in these cases it would be advisable to compare results to tools with negligible missing data. In particular, we would recommend CADD be included by default in variant prioritisation pipelines as it performed well when assessed on all variants (Supplementary Figure 12).

It is clear that recommendations made by the American College of Medical Genetics and Genomics (ACMG)^27^ are well founded, whereby in silico prediction tools are not sophisticated enough to be used in isolation for clinical diagnoses. Nevertheless, we find utility in the inclusion of such tools in variant prioritisation pipelines and would recommend the top performers found in this study (FATHMM, M-CAP, MetaLR, MetaSVM and VEST3). This aligns with ACMG advice, where consultation of predictions from more than one tool is generally preferred given the often found discrepancies in prediction between tools. Looking forward, performance of such tools can only improve given the rapidly increasing amount of data available for training classifiers and active development in the field. We are confident that major advances are now achievable and foresee a time where variant prioritisation tools will be elevated to use in clinical settings, contributing to the model of precision medicine.

## Methods

To assess performance of variant prioritisation tools by disease phenotype we developed an automated pipeline to integrate phenotypes with annotated variants. This pipeline allows us to update the benchmark data set with ease when new causative variants are discovered. Each component of the pipeline is fully described below:

### Human phenotype ontology

The HPO provides standardised terms to describe disease phenotypes.^18^ For our study the HPO allows us to separate diseases into a fixed number of classes based on phenotype. We used package ontologyIndex^28^ within R 3.2.0^29^ to read in the HPO obo file which was downloaded from http://human-phenotype-ontology.github.io/downloads.html on the 13th of January 2017. The HPO contains disease phenotypes under the umbrella term ‘Phenotypic abnormality’ (HP:0000118). We retrieved all 11,722 descendant terms of the ‘Phenotypic abnormality’ term using the get_descendants() function of the ontologyIndex package. Two examples of child terms of ‘Phenotypic abnormality’ include ‘Abnormality of the skeletal system’ and ‘Abnormality of the immune system’.

### Linking disease phenotypes to genes using Phenolyzer

Phenolyzer is a tool linking individual (or multiple) phenotypic terms to candidate genes.^19^ Here we use this tool to generate gene lists for all 11,722 HPO terms obtained above. We used the command line version available at https://github.com/WGLab/phenolyzer ensuring that we generated the same result as the Phenolyzer web server with default settings (i.e., options –p -ph -logistic -addon DB_DISGENET_GENE_DISEASE_SCORE,DB_GAD_GENE_DISEASE_SCORE -addon_weight 0.25). Phenolyzer matches each term to disease databases (Disease Ontology,^30^ CTD Medic vocabulary,^31^ HPO,^32^ OMIM synonyms,^33^ OMIM descriptors and Phenolyzer’s compiled disease vocabulary) and generates gene lists by using the resultant disease name(s) to query databases describing gene–disease associations (OMIM,^33^ Orphanet,^34^ ClinVar,^35^ Gene Reviews^36^ and GWAS Catalog.^37^) A score is assigned to each gene in the list reflecting the evidence for the gene–disease association. Gene scores in each list are normalised by dividing all scores by the maximum score. This results in scores ranging between 0 and 1 where higher scores indicate greater confidence. We refer to these lists as gene panels.

Phenolyzer can expand the aforementioned gene panels by including additional genes that are related to genes in the panel. Gene-gene relationships are determined from four databases (Human Protein Reference Database,^38^ NCBI’s BioSystems,^39^ HGNC Gene Family^40^ and Human Transcriptional Regulation Interactions database.^41^) A confidence score is assigned to each additional gene, combining the strength of association with genes in the panel and their confidence score. This ensures that related genes associated with top scoring panel genes have higher scores than related genes associated with lower scoring panel genes. Panel genes and related genes are renormalised as described above to produce the final prioritised gene panel for each disease. We refer to these lists as extended gene panels.

We assessed similarity between pairs of gene panels across HPO terms using the Bioconductor ‘GeneOverlap’ package.^42^ Similarity is based on the Jacard index which is calculated by dividing the number of intersections by the number of unions:

$$J\left( {{\rm GeneSet_ A},{\rm GeneSet_B}} \right) = \frac{{\left| {{\rm GeneSet_A}\mathop { \cap }\nolimits {\rm GeneSet_ B}} \right|}}{{\left| {{\rm GeneSet_A\mathop { \cup }\nolimits GeneSet_B}} \right|}}$$

The index ranges between 0 and 1, where 0 would indicate no similarity and 1 would indicate that the two lists are identical.

### Linking candidate genes to causative variants using dbNSFP annotations

The database for non-synonymous SNPs’ functional predictions (dbNSFP) contains annotation for genes and 83,422,341 potential nsSNVs in the human genome.^21,43^ We used dbNSFP version 3.3a (release 30 November, 2016) which is based on Gencode release 22/Ensembl version 79.^44,45^ We selected all variants occurring in any of the candidate gene lists generated by Phenolyzer.

dbNSFP includes ClinVar^20^ annotation (version 20161101) describing the pathogenicity of variants implicated in Mendelian disorders. ClinVar uses the five clinical significance categories recommended by the ACMG^27^ (benign, likely benign, uncertain significance, likely pathogenic and pathogenic). We restricted our analysis to the “pathogenic” category. In total, we obtained 24,792 pathogenic variants linked to genes associated with human disease phenotypes.

We used dbNSFP gene annotation to investigate properties of the genes returned by Phenolyzer. Specifically, we used predicted haploinsufficiency of genes,^23^ predicted probability of recessive disease causation,^9^ RVIS^24^ and LoFtool gene intolerance scores.^25^

### Benign variants

We selected a set of 5756 benign variants from the ‘VariBenchSelected’ data set made available by Grimm et al.^14^ (downloaded from http://structure.bmc.lu.se/VariBench/GrimmDatasets.php on the 11th of March 2016) and annotated these variants using dbNSFP.

### Performance evaluation

We evaluated the performance of variant prioritisation tools by assessing their ability to discriminate ClinVar pathogenic variants from benign variants. Assessments were performed for each HPO term, based on dbNSFP annotated variants from different types of Phenolyzer gene panels. In total we used six such panels for each term using normalised confidence score thresholds of 0, 0.25 or 0.5 for both gene panels and extended gene panels. Furthermore, we assessed performance using all variants or the subset of variants with no missing scores for the tools tested here (i.e., a complete case analysis). The same set of 5756 benign variants was used for each test.

We included 18 functional prediction tools in our study: SIFT,^46^ PROVEAN,^47^ PolyPhen2 (HDIV and HVAR),^48^ LRT,^49^ MutationTaster,^50^ MutationAssessor,^51^ FATHMM,^52^ fathmm-MKL,^53^ CADD,^54^ VEST3,^55^ fitCons-i6,^56^ DANN,^57^ MetaSVM,^12^ MetaLR,^12^ GenoCanyon,^58^ Eigen-PC^59^ and M-CAP^60^ and 6 conservation based tools: phyloP (100way_vertebrate and 20way_mammalian),^61^ phastCons (100way_vertebrate and 20way_mammalian),^62^ GERP++^63^ and SiPhy.^64^ We used the dbNSFP converted rank scores for each tool. The rank score is a transformation applied to the prediction scores for each tool, where firstly, scores were reverse coded as necessary so that increasing values of the score indicate increasing evidence of pathogenicity. Secondly, scores are ranked and divided by the total number of scores for that particular tool. This means that the rank score is restricted to be within the range of 0–1. Almost all genes have multiple transcript isoforms and variants can therefore have an effect on several transcripts. In such cases the highest score (i.e., most pathogenic) is assigned to the nsSNV.

We used R package PRROC^65^ to calculate the auROC and the auPRC based on the interpolation of Davis and Goadrich.^66^ These measures quantify the classification ability of each variant prioritisation tool. The aucpr.conf.int.expit() function available at https://github.com/kboyd/raucpr/blob/master/precision_recall.r was used to calculate 95% logit confidence intervals for each auROC and auPRC estimate.^67^ A true positive (TP) is considered to be a correctly predicted pathogenic variant, a false negative (FN) is a pathogenic variant predicted to be benign, a FP is a benign variant predicted to be pathogenic and a true negative (TN) is a correctly predicted benign variant. The auROC plots the TP rate (TPR) versus the FP rate (FPR) for differing cut points of the variant prioritisation tool score, whereas the auPRC plots precision (positive predictive value) versus recall (TPR). The TPR is TP/(TP + FN), the FPR is FP/(FP + TN) and precision is TP/(TP + FP). Perfect classification of variants would result in an auROC and an auPRC of 1, whereas random classification of variants would result in an auROC of 0.5 and an auPRC equal to the ratio of pathogenic to benign variants. The aheatmap() function of the R NMF package^68^ was used to produce heatmaps of auROC and auPRC values.

In diagnostic labs, variant prioritisation generally involves identifying a small number of pathogenic variants from a larger number of benign variants. Our data set mimics this situation for most HPO terms because we assign the same set of 5756 benign variants to each term (this number reduced to 2910 for the complete case analysis). For this task, the auPRC is a more informative measure of performance than the auROC,^69^ because it better quantifies the number of FPs. The auROC plots the TPR versus the FPR and the FPR remains low even when there are many FPs, due to the majority of benign variants being correctly classified. The auPRC plots precision versus the TPR and precision gives a more accurate picture of the number of FPs when compared to the FPR, because precision only considers variants that are predicted to be pathogenic. A hypothetical example can illustrate this point; if we have 100 pathogenic variants and 5000 benign variants and consider a particular cut point resulting in 85 TPs, 15 FNs, 500 FPs and 4500 TNs then the TPR is 0.85 (85/100) and the FPR is 0.1 (500/5000). Though the FPR appears to be quite low, the ratio of FPs to TPs is large (500:85), meaning that we will be following up ~6 non-informative variants for every pathogenic variant. For this hypothetical example, precision is 0.15 (85/585) which means that only 15% of the variants predicted to be pathogenic actually are pathogenic. Reducing the number of variants to follow up is important for clinical decision making.

We produced HTML tables of the performance results using the R package DT.^70^ These tables can be easily searched and filtered for HPO terms of interest.

### Code availability

Code used to generate results for this study is available as Supplementary Files 3 and 4.

### Data availability

The data that support the findings of this study are available from the corresponding author upon reasonable request.

## Supplementary material

Supplementary Figures 1 to 21

Supplementary Table 1

Supplementary Table 2

Supplementary Table 3

Supplementary Table 4

Supplementary Table 5

Supplementary File 1

Supplementary File 2

Supplementary File 3

Supplementary File 4

## Electronic supplementary material

**Supplementary information** accompanies the paper on the *npj Genomic Medicine* website (10.1038/s41525-018-0044-9).
