# Supplementary material for: A phenotype centric benchmark of variant prioritisation tools
Source: NPJ Genom Med. 2018 Feb 5;3:5. doi: 10.1038/s41525-018-0044-9 (PMC5799157; doi:10.1038/s41525-018-0044-9)
Supplement: Supplementary file 1 — Supplementary Figures 1 to 21 [file 41525_2018_44_MOESM1_ESM.pdf]

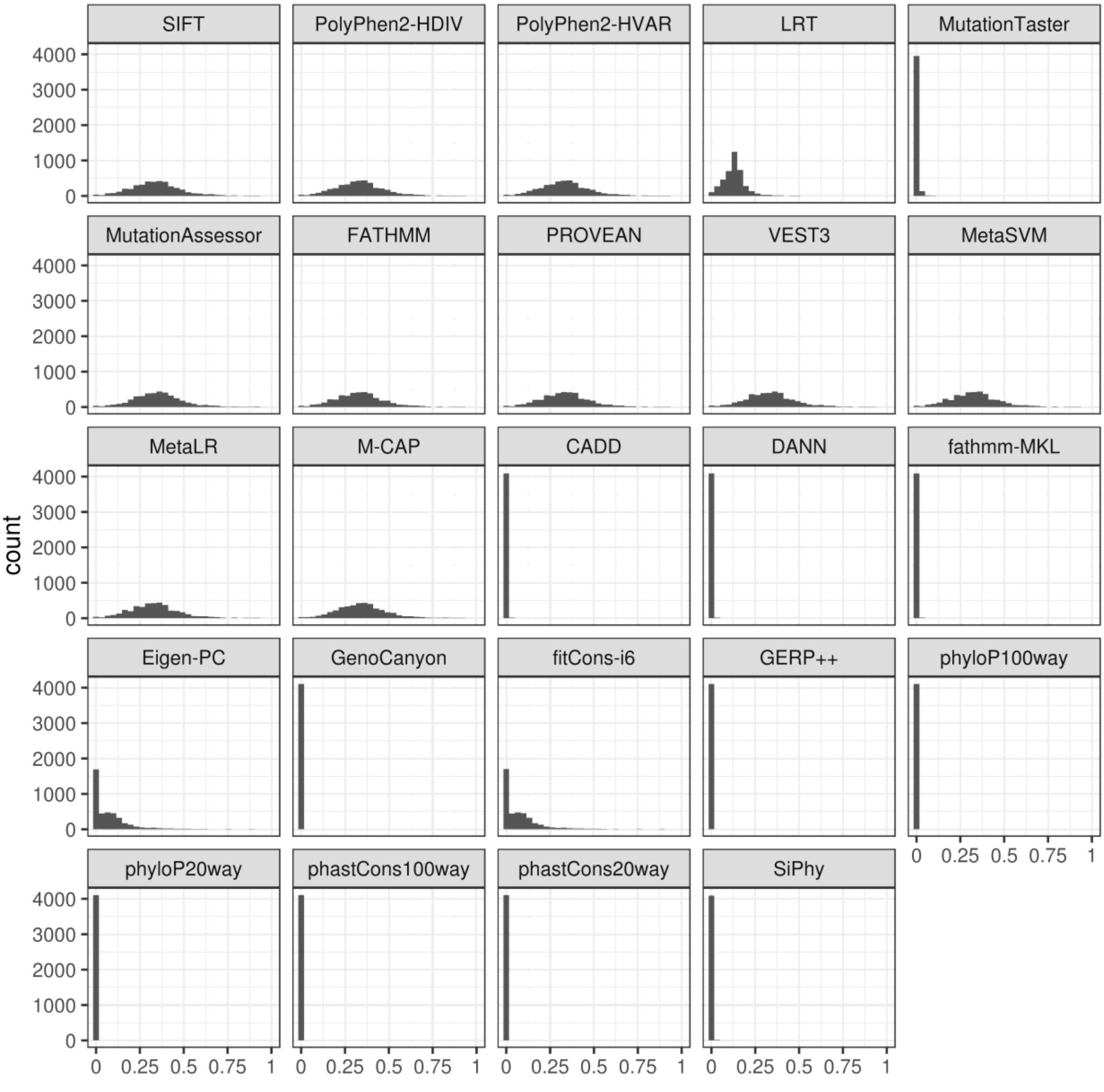

Figure S1: Distribution of the proportion of pathogenic variants with missing scores across the 4,108 HPO *Phenotypic abnormality* terms for the 24 variant prioritisation tools

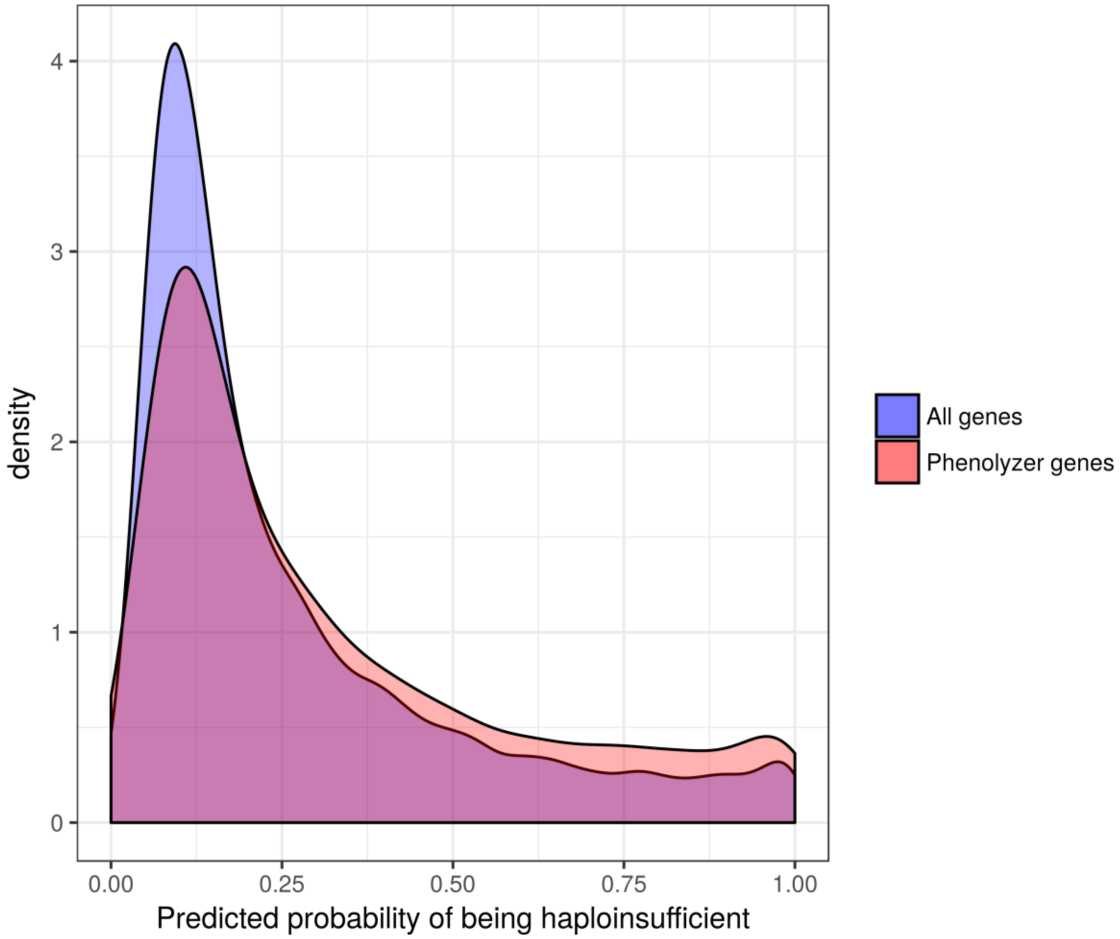

Figure S2: Distribution of the estimated probability of gene haploinsufficiency for all genes (n=17,082) and for Phenolyzer genes (n=4,679)

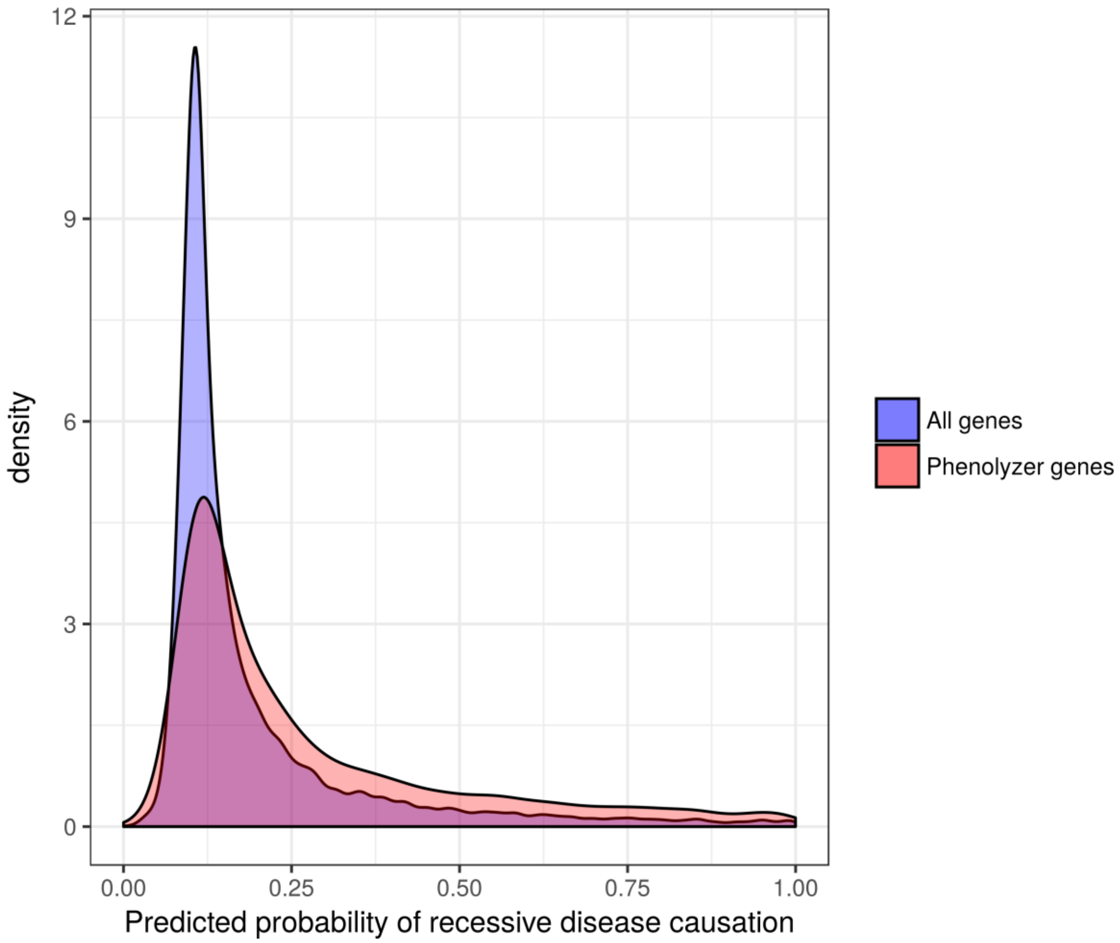

Figure S3: Distribution of the estimated probability of recessive disease causation for all genes (n=14,142) and for Phenolyzer genes (n=4,338)

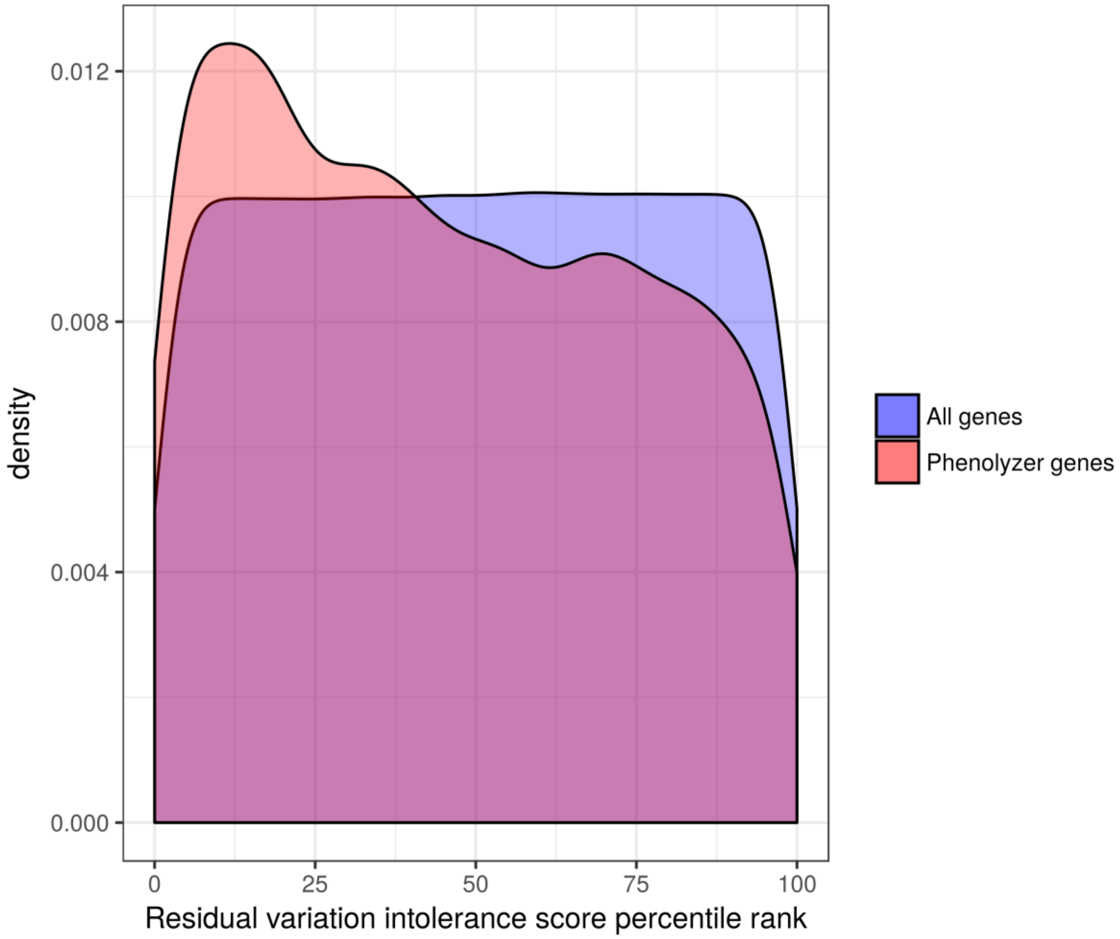

Figure S4: Distribution of the residual variation intolerance score percentile rank for all genes (n=16,956) and for Phenolyzer genes (n=4,774)

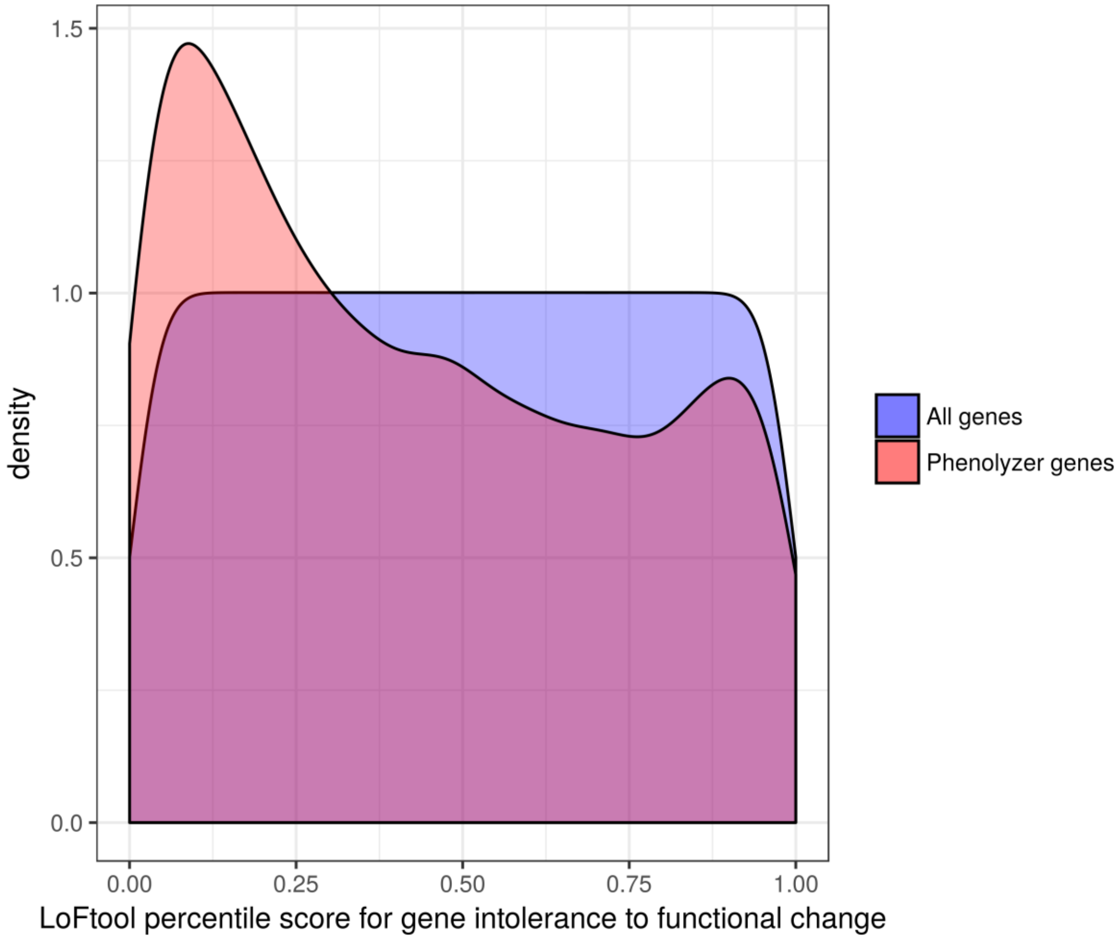

Figure S5: Distribution of the LoFtool percentile score for all genes (n=14,515) and for Phenolyzer genes (n=4,285)

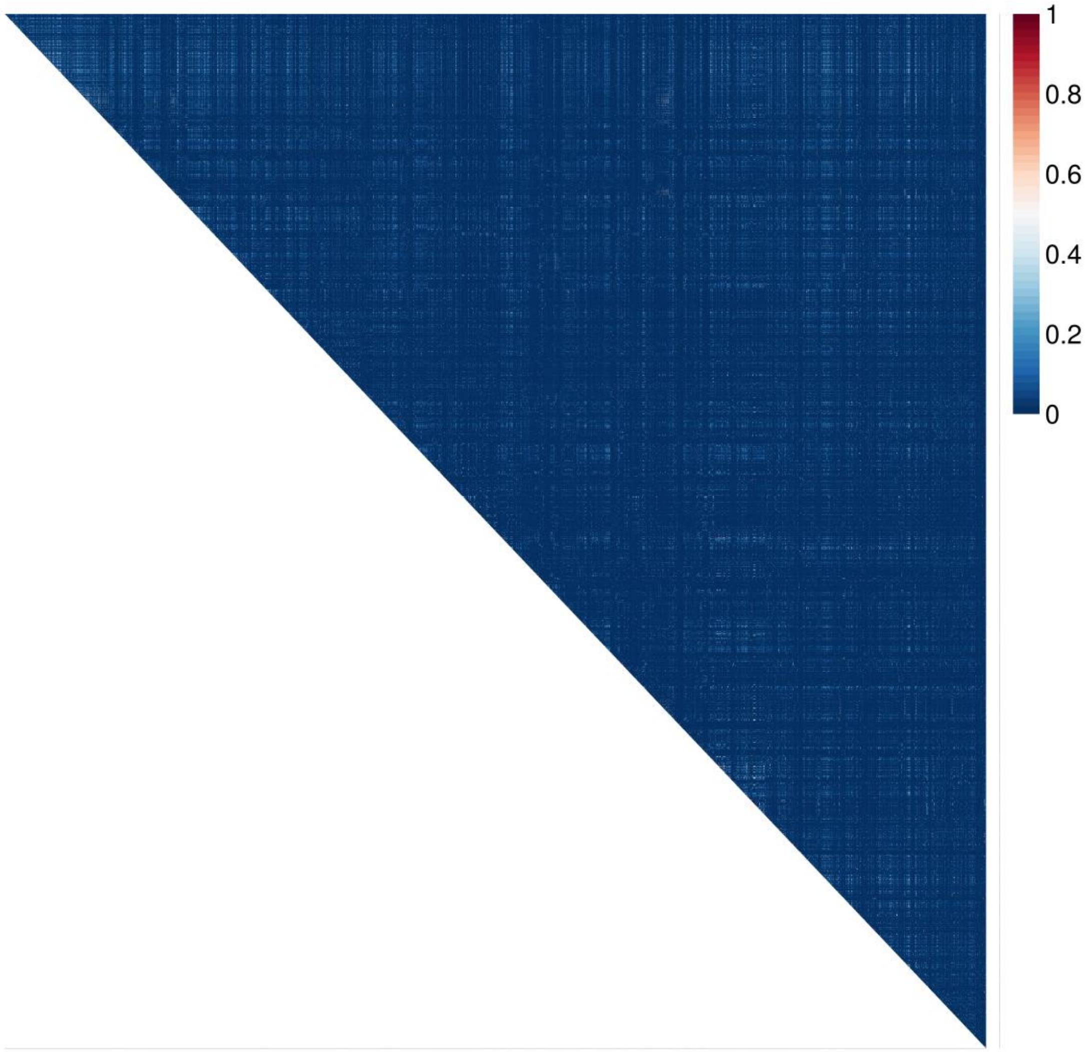

Figure S6: Heatmap of the Jacard index for all pairs of gene panels across the HPO terms used in the complete cases analysis (n=4,026)

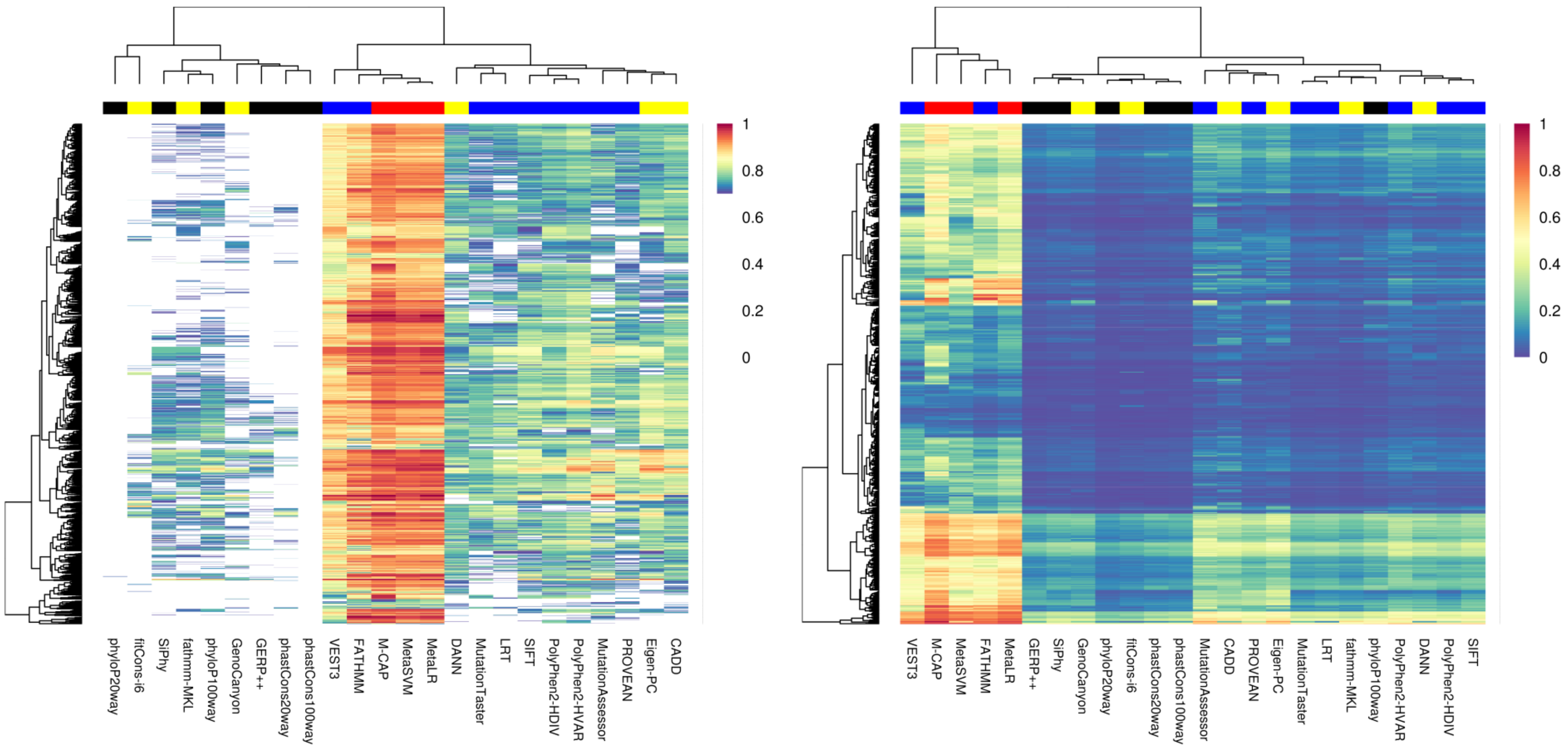

Figure S7: Heatmaps showing auROC (left) and auPRC (right) values for the 3,326 HPO *Phenotypic abnormality* terms when using Phenolyzer gene panels with a score threshold of 0.25. Colour coding of columns represents the score type for each variant prioritisation tool where black=conservation scores, red=ensemble scores, blue=functional prediction scores and yellow=general prediction scores. The heatmap colour scale of the auROC (left) values has been adjusted to highlight moderate to strong performance by only colour coding auROC values greater than or equal to 0.7.

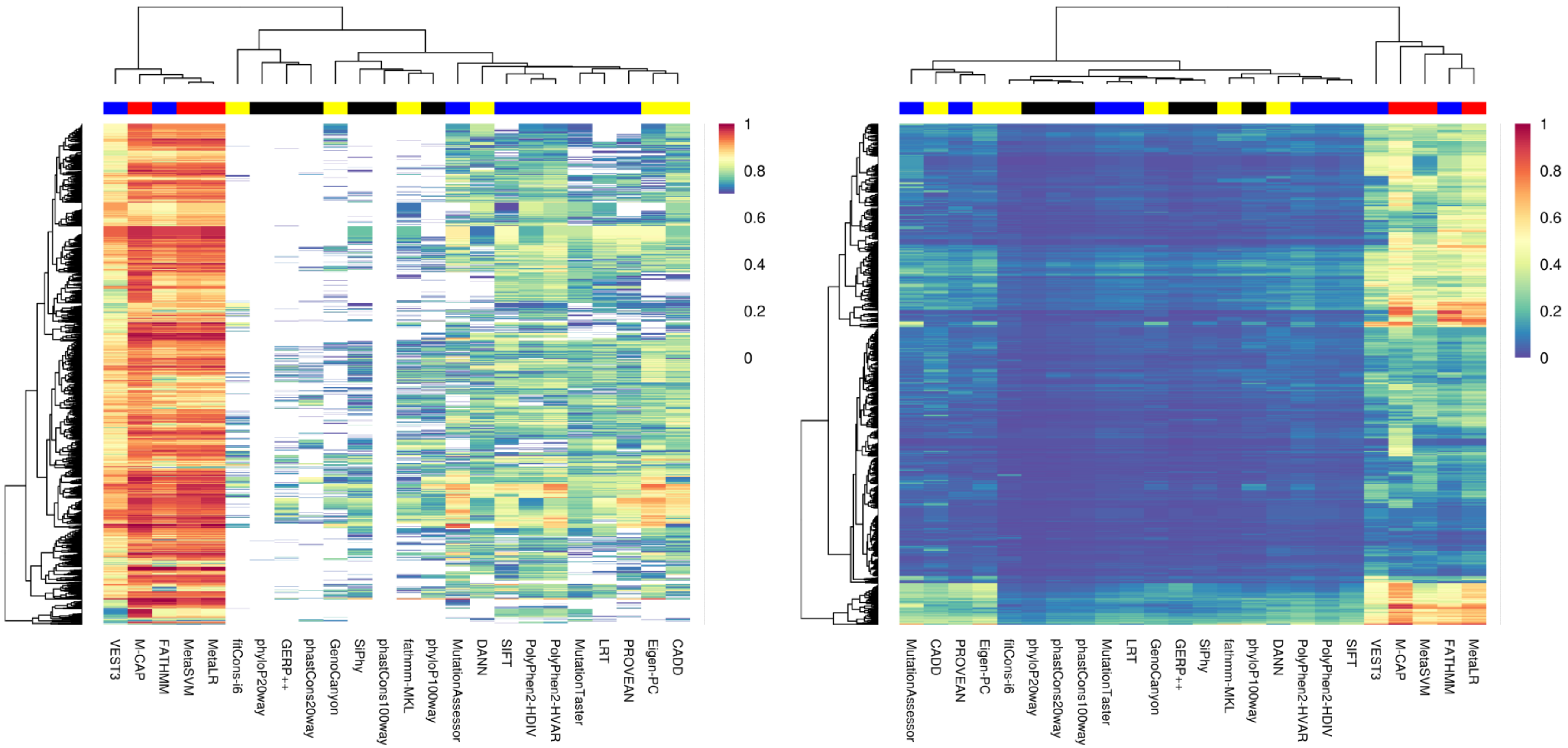

Figure S8: Heatmaps showing auROC (left) and auPRC (right) values for the 2,551 HPO *Phenotypic abnormality* terms when using Phenolyzer gene panels with a score threshold of 0.5. Colour coding of columns represents the score type for each variant prioritisation tool where black=conservation scores, red=ensemble scores, blue=functional prediction scores and yellow=general prediction scores. The heatmap colour scale of the auROC (left) values has been adjusted to highlight moderate to strong performance by only colour coding auROC values greater than or equal to 0.7.

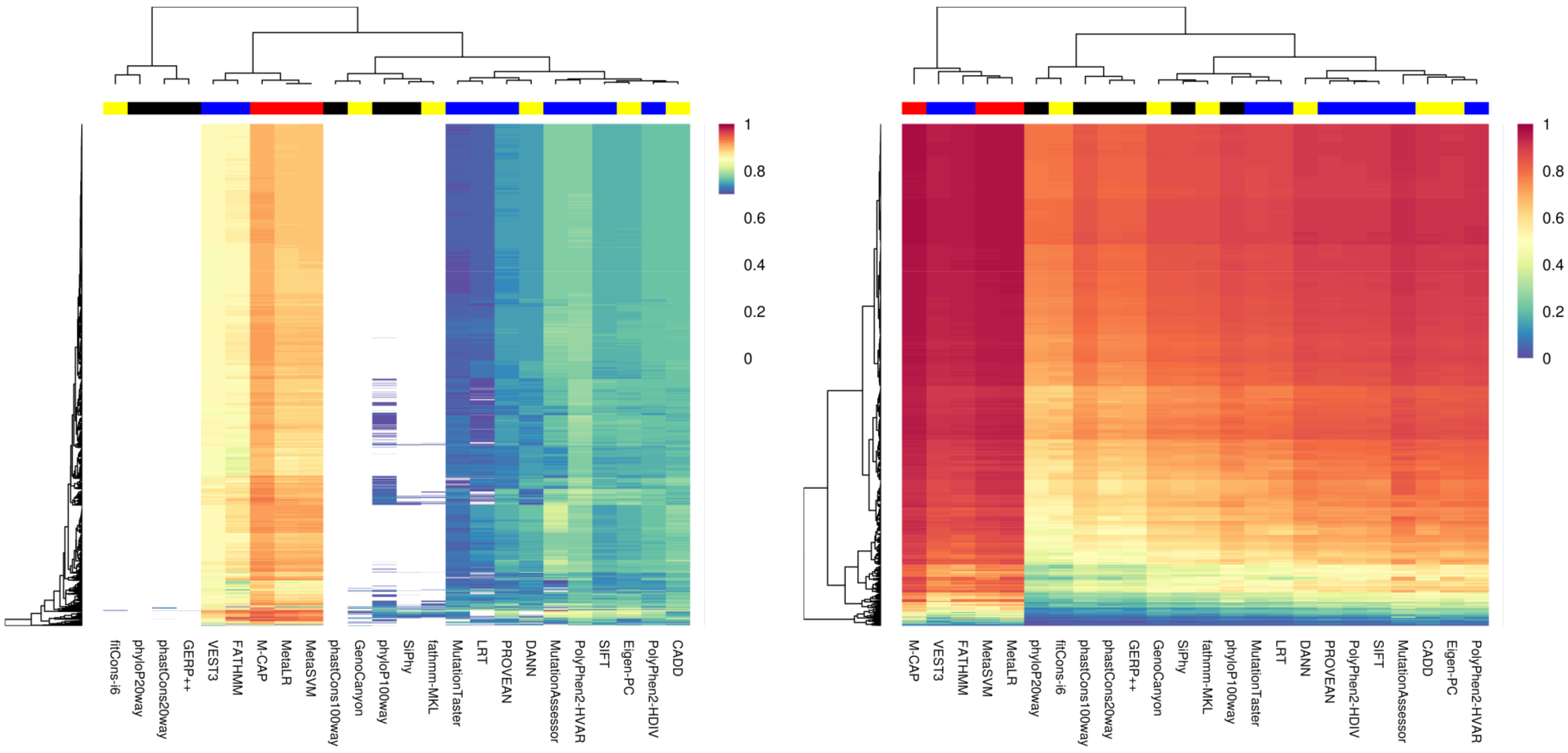

Figure S9: Heatmaps showing auROC (left) and auPRC (right) values for the 6,348 HPO *Phenotypic abnormality* terms when using Phenolyzer expanded gene panels with no score threshold. Colour coding of columns represents the score type for each variant prioritisation tool where black=conservation scores, red=ensemble scores, blue=functional prediction scores and yellow=general prediction scores. The heatmap colour scale of the auROC (left) values has been adjusted to highlight moderate to strong performance by only colour coding auROC values greater than or equal to 0.7.

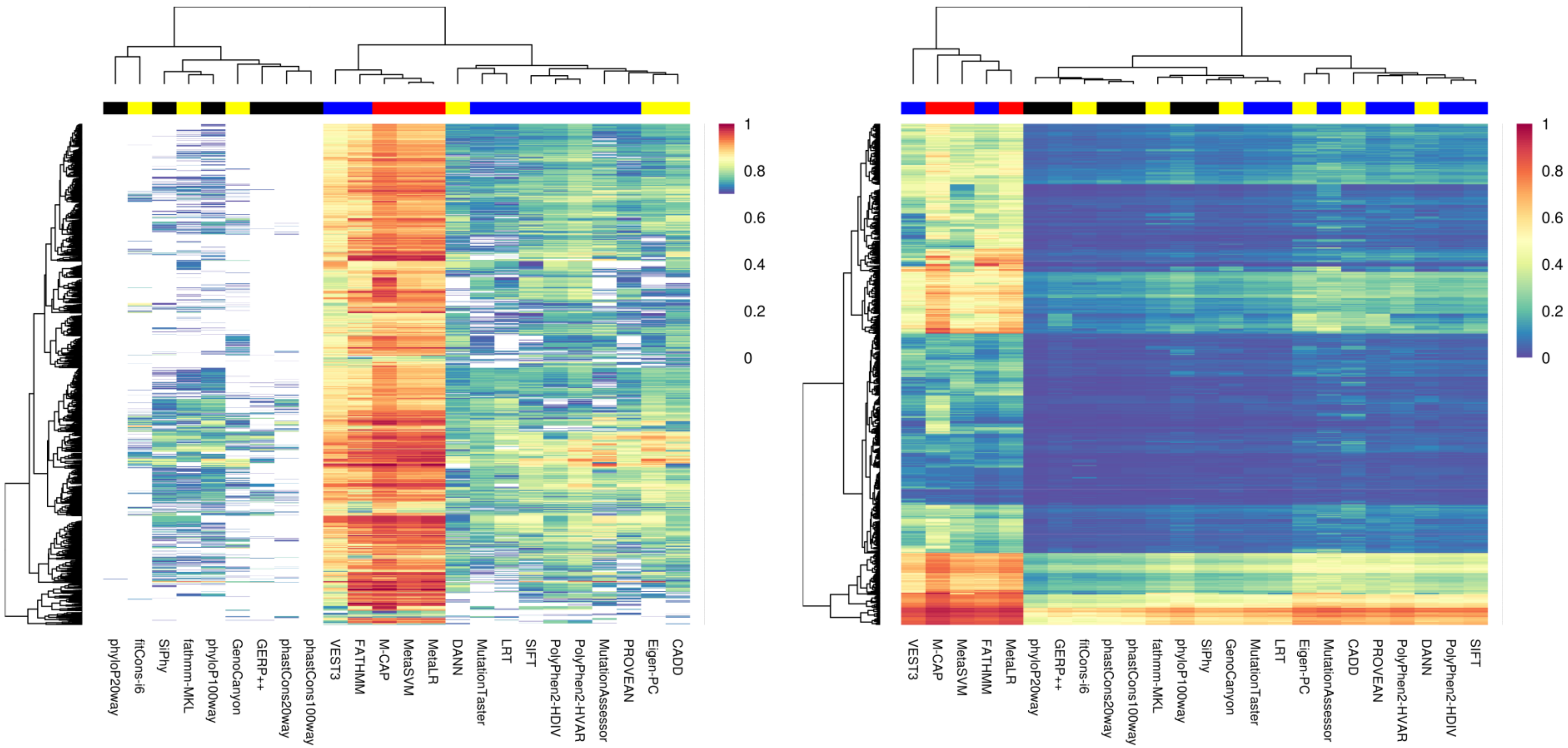

Figure S10: Heatmaps showing auROC (left) and auPRC (right) values for the 3,383 HPO *Phenotypic abnormality* terms when using Phenolyzer expanded gene panels with a score threshold of 0.25. Colour coding of columns represents the score type for each variant prioritisation tool where black=conservation scores, red=ensemble scores, blue=functional prediction scores and yellow=general prediction scores. The heatmap colour scale of the auROC (left) values has been adjusted to highlight moderate to strong performance by only colour coding auROC values greater than or equal to 0.7.

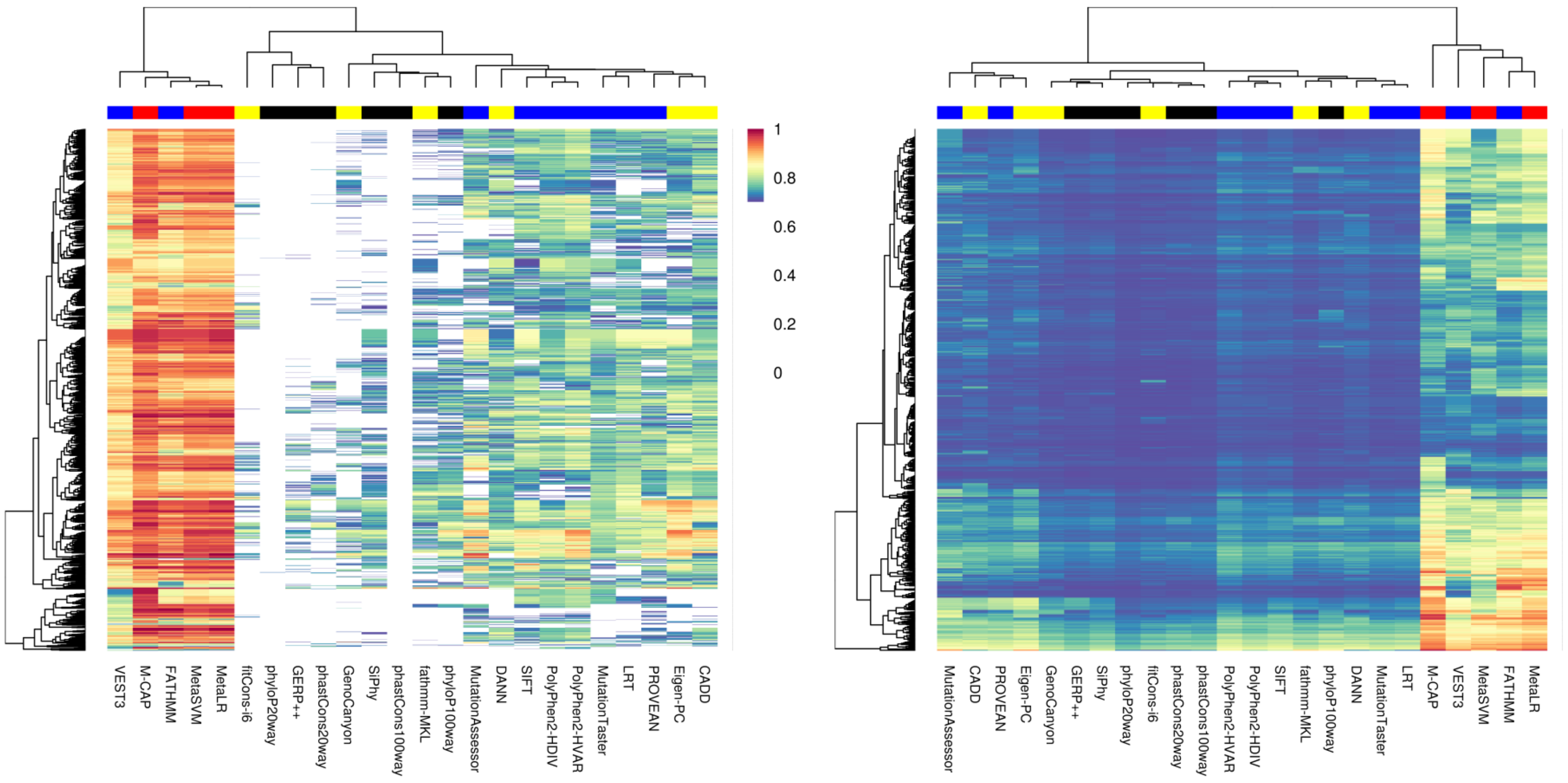

Figure S11: Heatmaps showing auROC (left) and auPRC (right) values for the 2,638 HPO *Phenotypic abnormality* terms when using Phenolyzer expanded gene panels with a score threshold of 0.5. Colour coding of columns represents the score type for each variant prioritisation tool where black=conservation scores, red=ensemble scores, blue=functional prediction scores and yellow=general prediction scores. The heatmap colour scale of the auROC (left) values has been adjusted to highlight moderate to strong performance by only colour coding auROC values greater than or equal to 0.7.

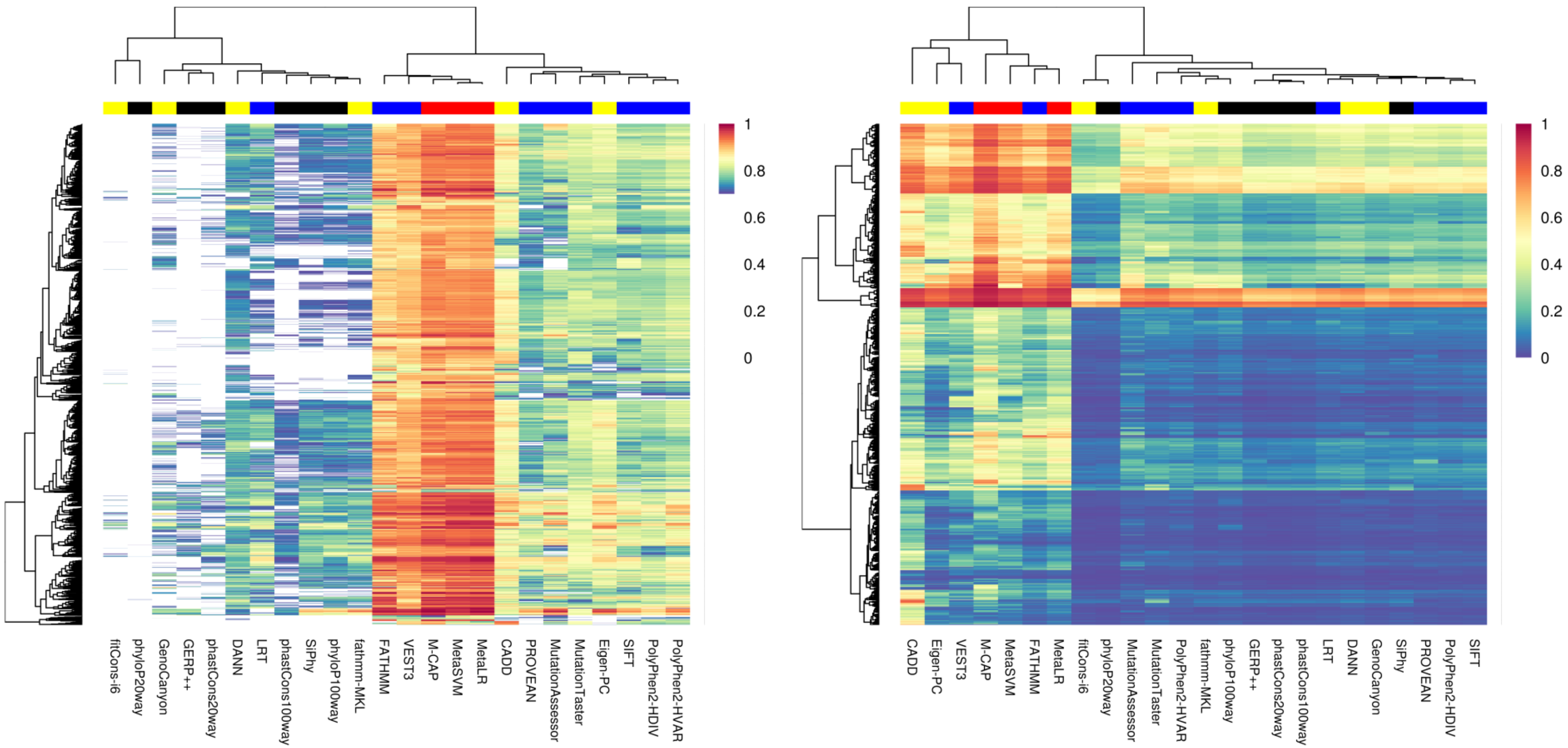

Figure S12: Heatmaps showing auROC (left) and auPRC (right) values for the 4,108 HPO *Phenotypic abnormality* terms when using Phenolyzer gene panels with no score threshold. Colour coding of columns represents the score type for each variant prioritisation tool where black=conservation scores, red=ensemble scores, blue=functional prediction scores and yellow=general prediction scores. The heatmap colour scale of the auROC (left) values has been adjusted to highlight moderate to strong performance by only colour coding auROC values greater than or equal to 0.7.

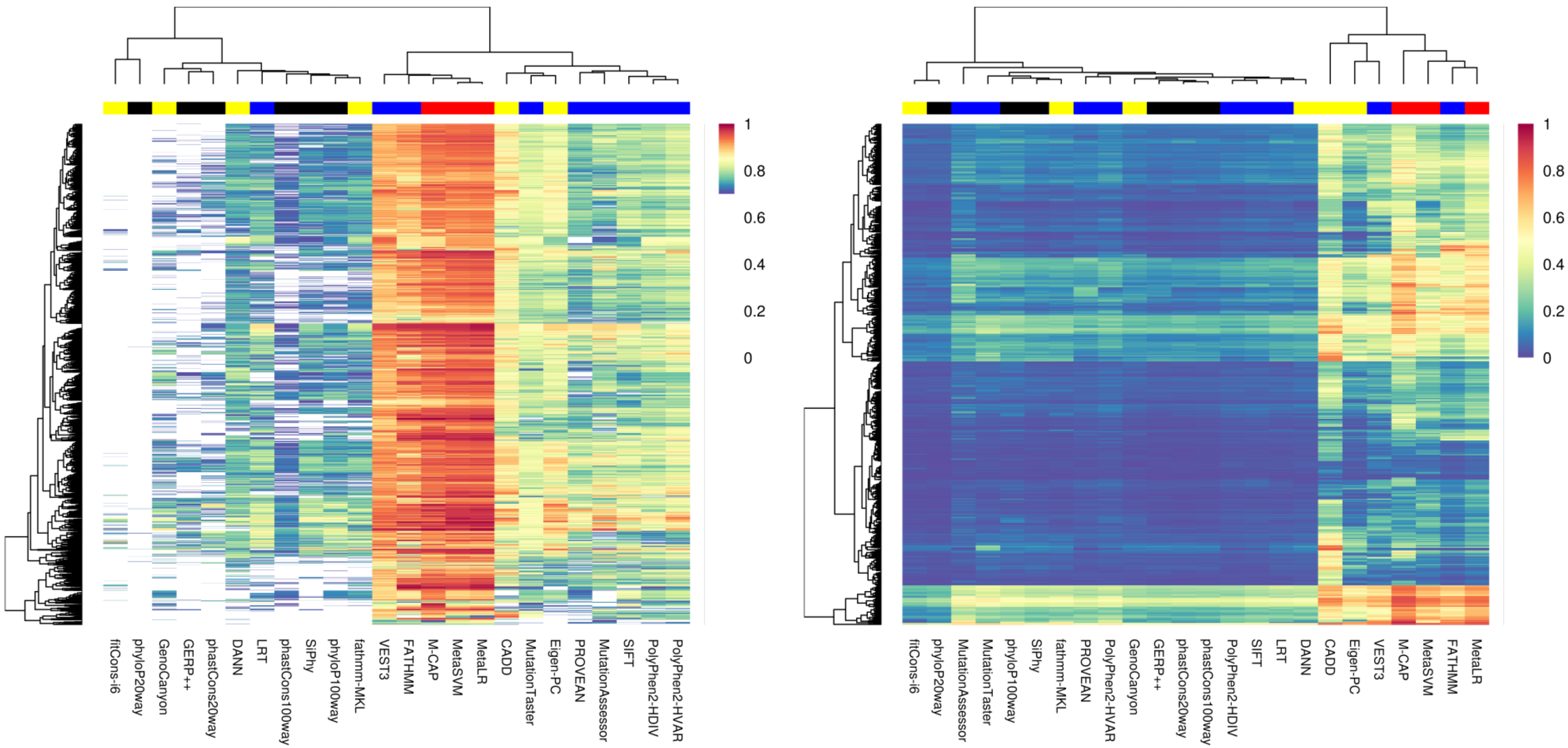

Figure S13: Heatmaps showing auROC (left) and auPRC (right) values for the 3,444 HPO *Phenotypic abnormality* terms when using Phenolyzer gene panels with a score threshold of 0.25. Colour coding of columns represents the score type for each variant prioritisation tool where black=conservation scores, red=ensemble scores, blue=functional prediction scores and yellow=general prediction scores. The heatmap colour scale of the auROC (left) values has been adjusted to highlight moderate to strong performance by only colour coding auROC values greater than or equal to 0.7.

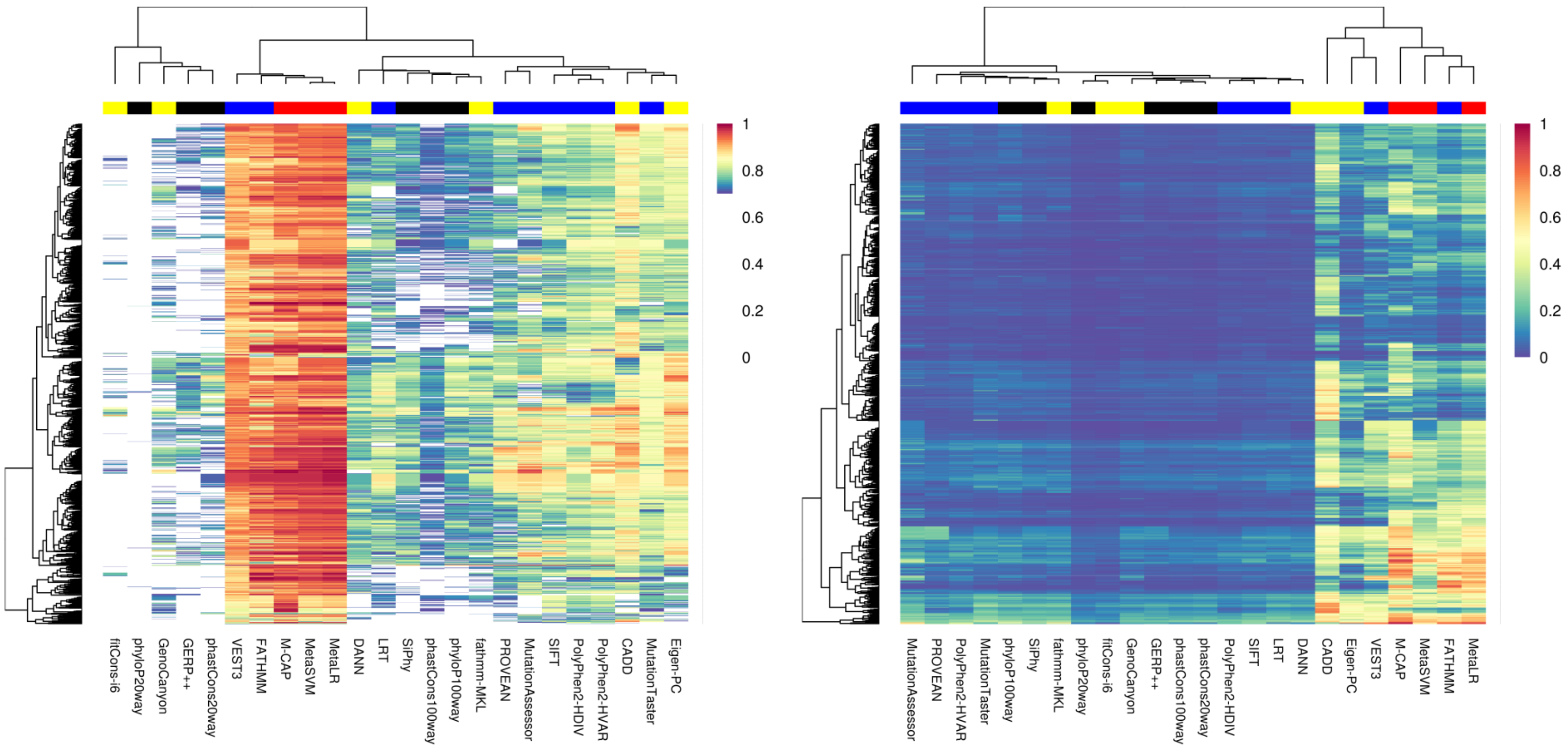

Figure S14: Heatmaps showing auROC (left) and auPRC (right) values for the 2,735 HPO *Phenotypic abnormality* terms when using Phenolyzer gene panels with a score threshold of 0.5. Colour coding of columns represents the score type for each variant prioritisation tool where black=conservation scores, red=ensemble scores, blue=functional prediction scores and yellow=general prediction scores. The heatmap colour scale of the auROC (left) values has been adjusted to highlight moderate to strong performance by only colour coding auROC values greater than or equal to 0.7.

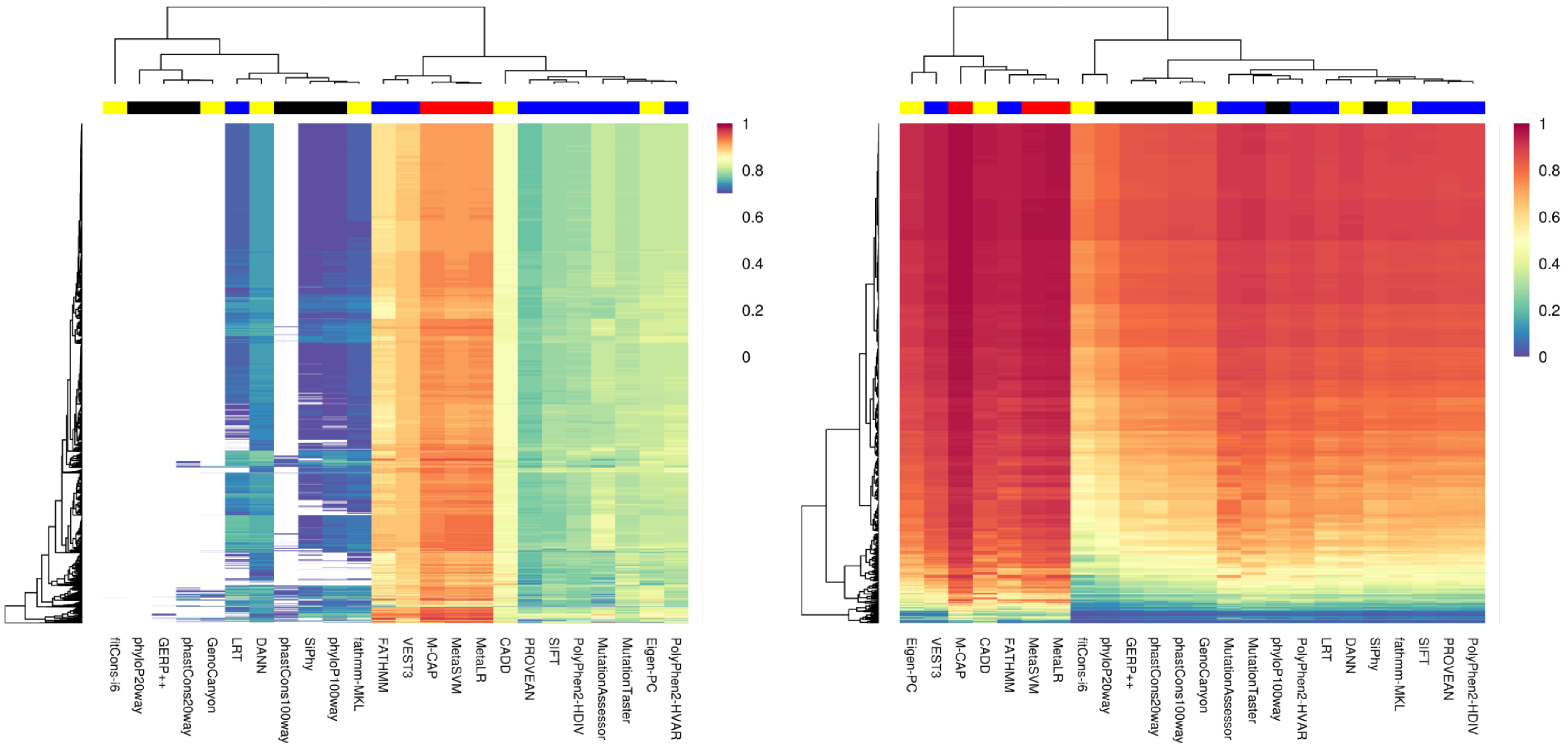

Figure S15: Heatmaps showing auROC (left) and auPRC (right) values for the 6,379 HPO *Phenotypic abnormality* terms when using Phenolyzer expanded gene panels with no score threshold. Colour coding of columns represents the score type for each variant prioritisation tool where black=conservation scores, red=ensemble scores, blue=functional prediction scores and yellow=general prediction scores. The heatmap colour scale of the auROC (left) values has been adjusted to highlight moderate to strong performance by only colour coding auROC values greater than or equal to 0.7.

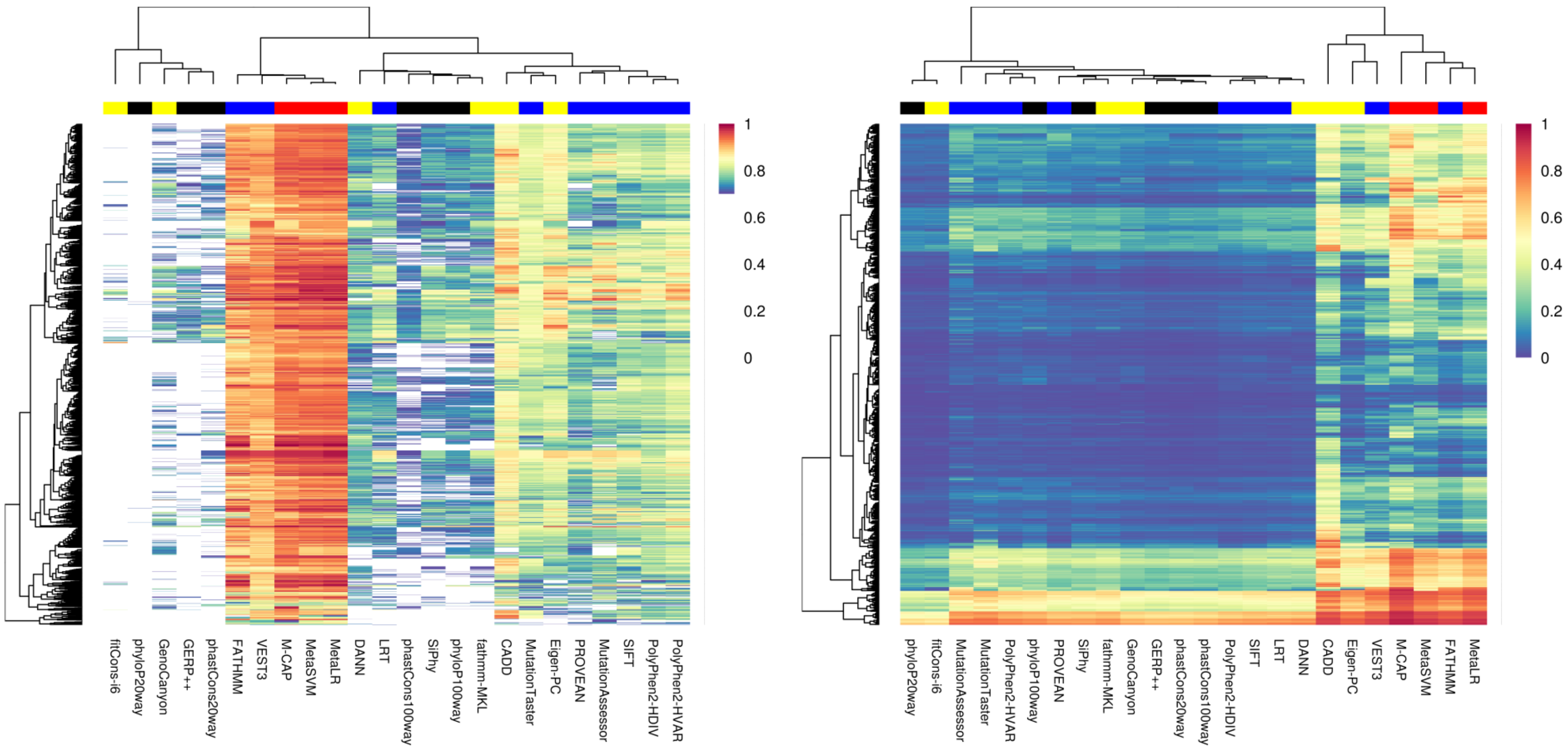

Figure S16: Heatmaps showing auROC (left) and auPRC (right) values for the 3,497 HPO *Phenotypic abnormality* terms when using Phenolyzer expanded gene panels with a score threshold of 0.25. Colour coding of columns represents the score type for each variant prioritisation tool where black=conservation scores, red=ensemble scores, blue=functional prediction scores and yellow=general prediction scores. The heatmap colour scale of the auROC (left) values has been adjusted to highlight moderate to strong performance by only colour coding auROC values greater than or equal to 0.7.

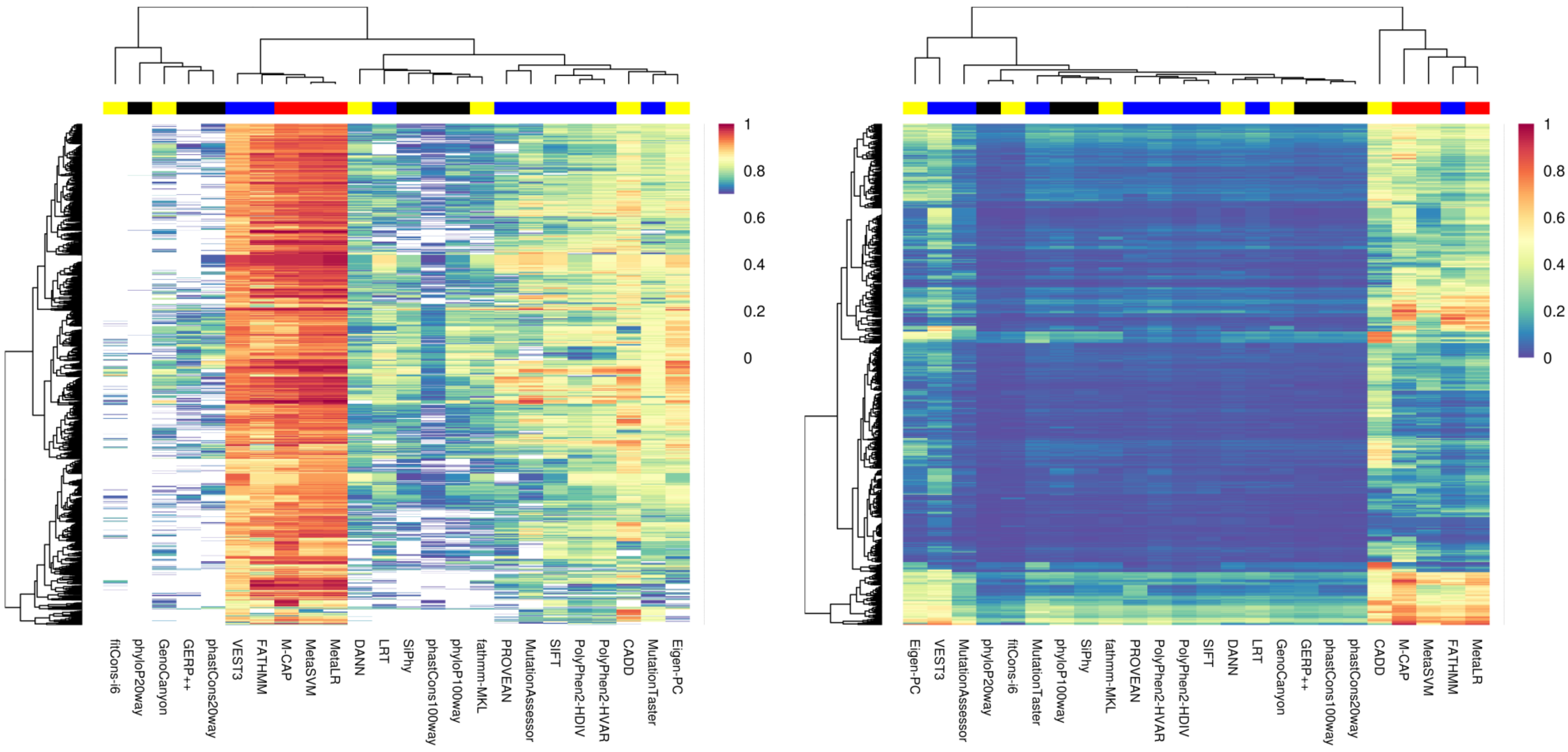

Figure S17: Heatmaps showing auROC (left) and auPRC (right) values for the 2,824 HPO *Phenotypic abnormality* terms when using Phenolyzer expanded gene panels with a score threshold of 0.5. Colour coding of columns represents the score type for each variant prioritisation tool where black=conservation scores, red=ensemble scores, blue=functional prediction scores and yellow=general prediction scores. The heatmap colour scale of the auROC (left) values has been adjusted to highlight moderate to strong performance by only colour coding auROC values greater than or equal to 0.7.

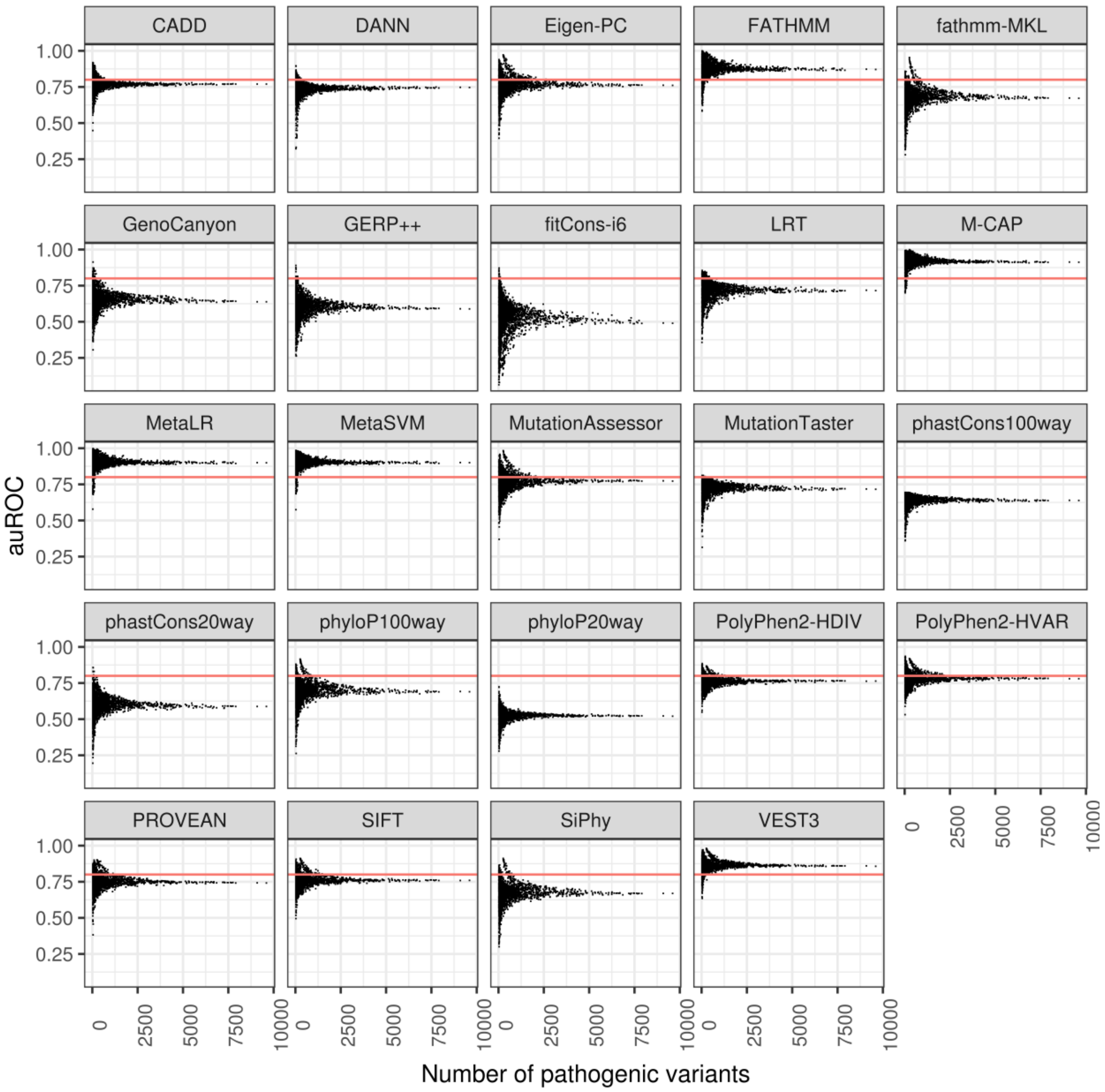

Figure S18: Performance (auROC) versus number of ClinVar pathogenic variants across all tested variant prioritisation tools. The horizontal red line is equal to 0.8 and indicates strong performance.

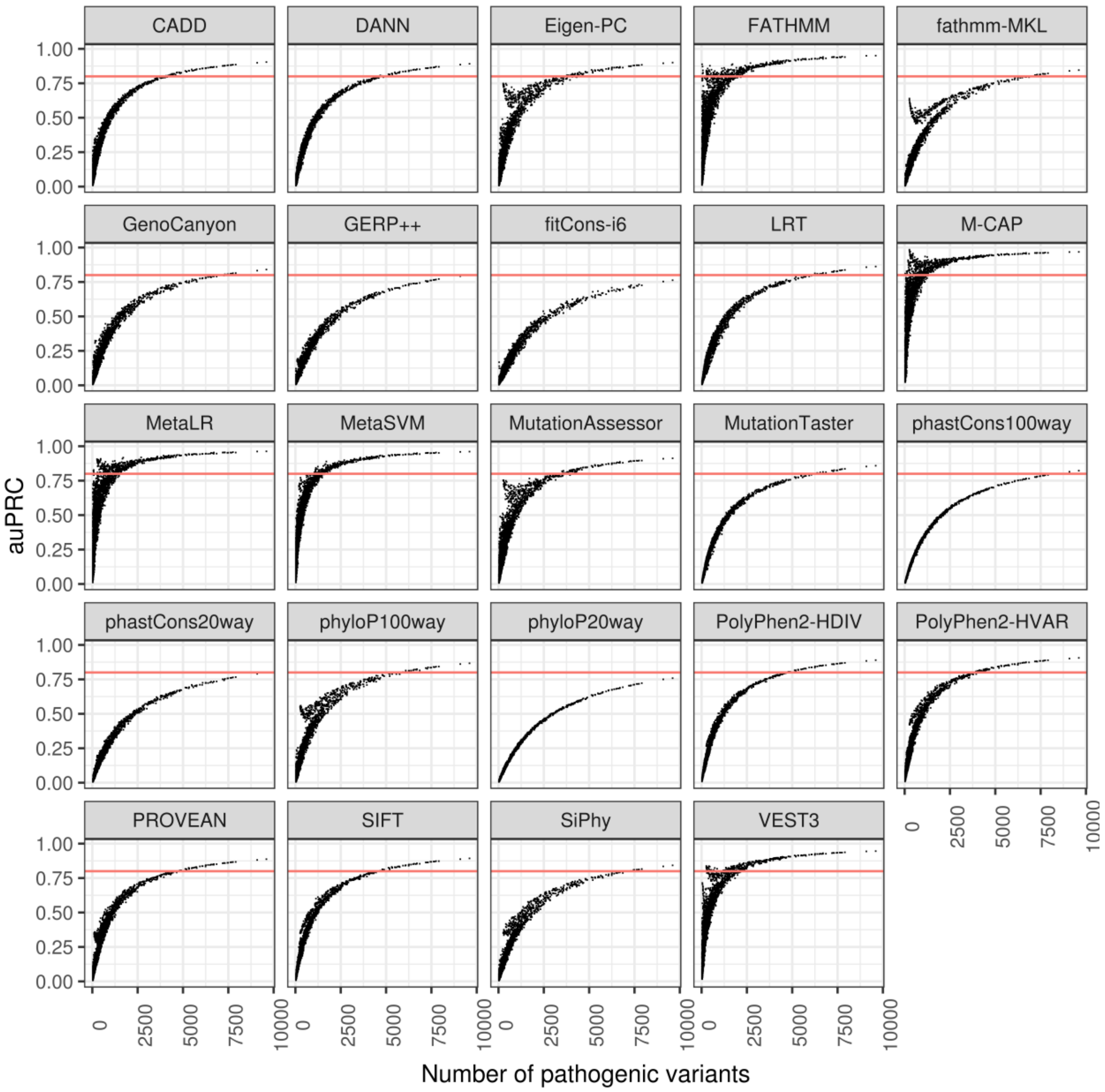

Figure S19: Performance (auPRC) versus number of ClinVar pathogenic variants across all tested variant prioritisation tools. The horizontal red line is equal to 0.8 and indicates strong performance.

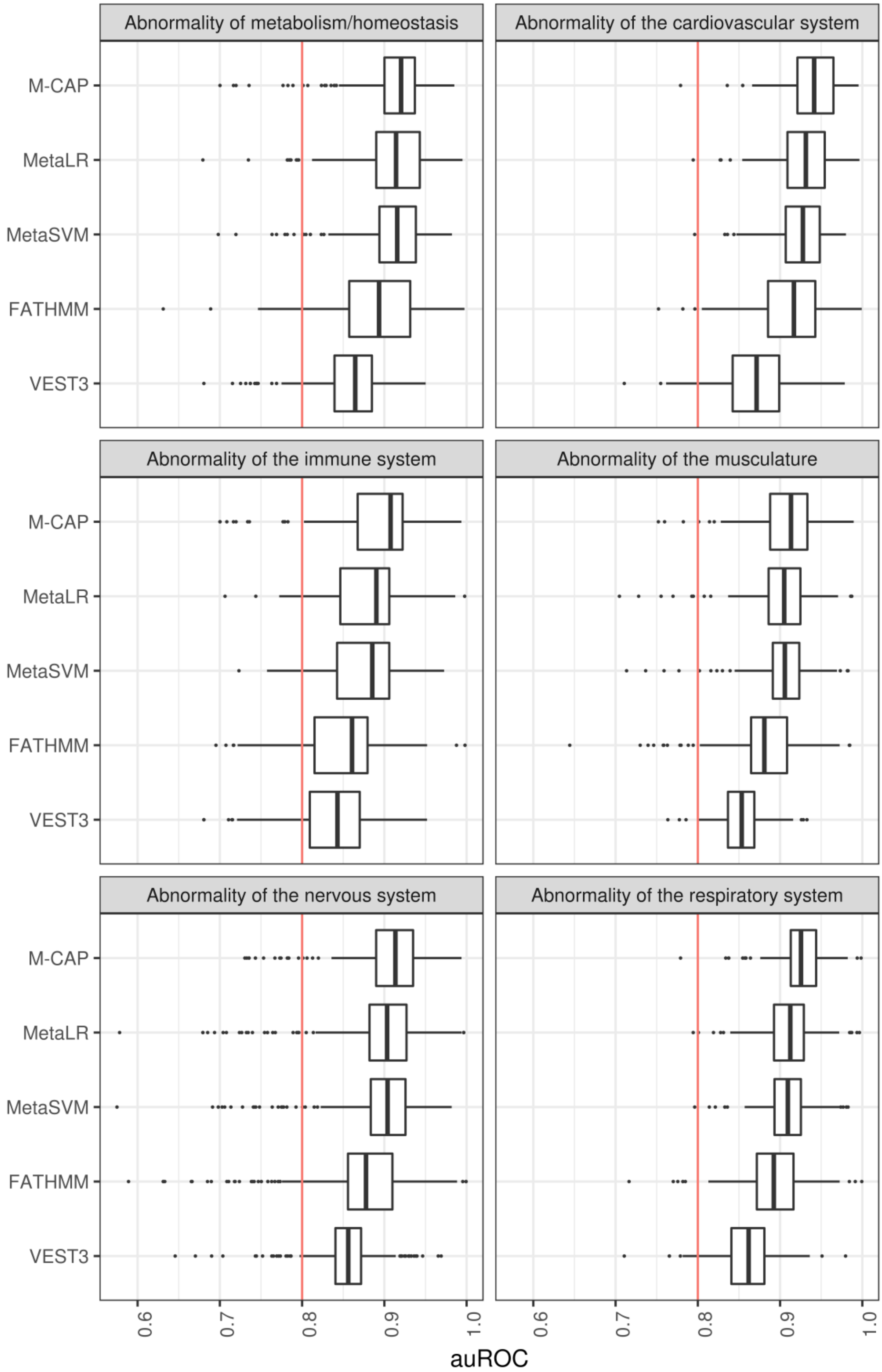

Figure S20: Boxplots showing the auROC values across the top performing variant prioritisation tools for selected HPO *Phenotypic abnormality* terms. The vertical red line indicates a strong performance value of 0.8.

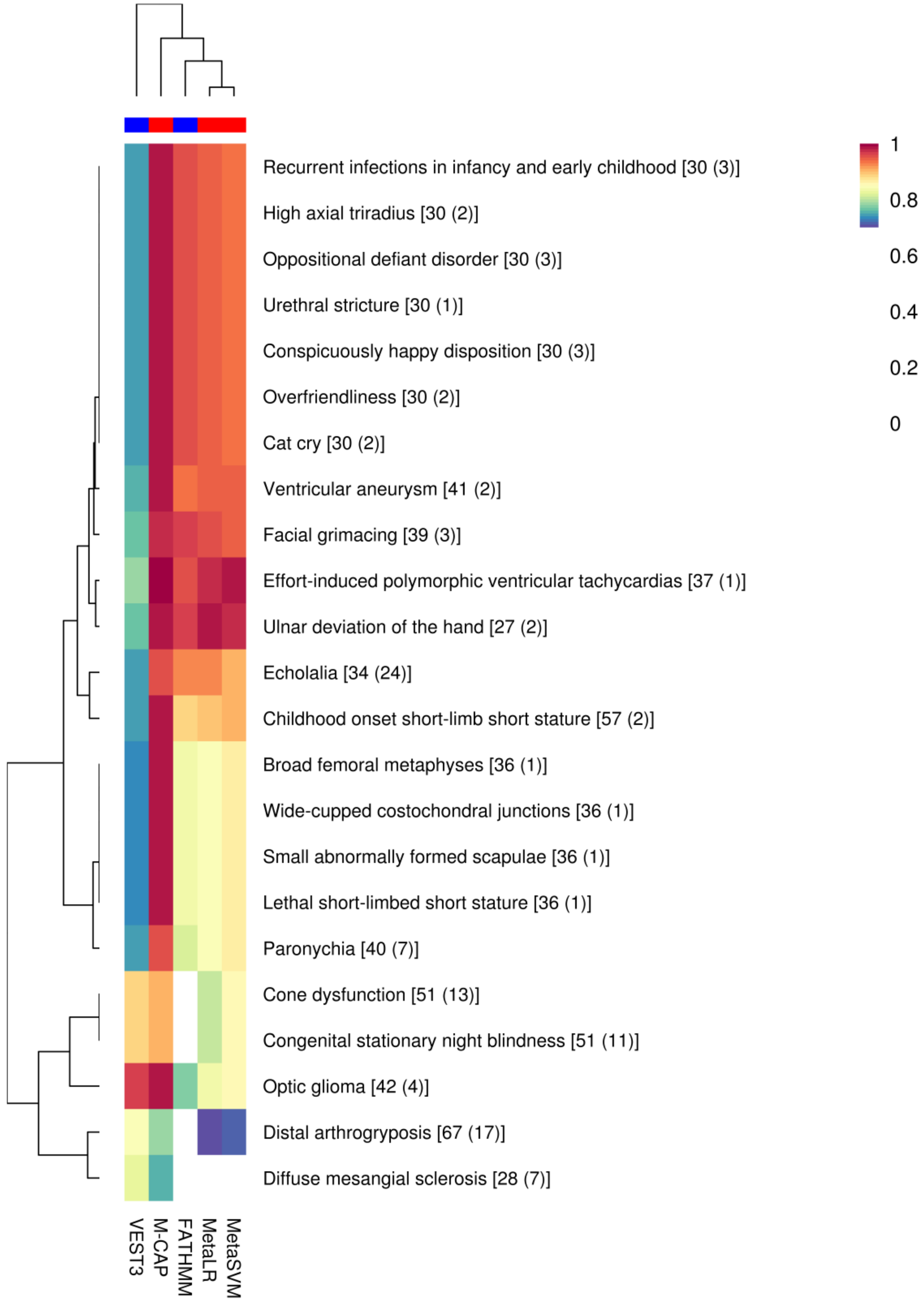

Figure S21: Heatmap showing auROC for HPO *Phenotypic abnormality* terms where top performing variant prioritisation tools differ by greater than 0.2. Colour coding of columns represents the score type for each variant prioritisation tool where red=ensemble scores and blue=functional prediction scores. Row annotation includes term and [Number of ClinVar pathogenic variants in dbNSFP (Number of seed genes returned by Phenolyzer)].
